# Supplementary material for: Apolar chemical environments compact unfolded RNAs and can promote folding
Source: Biophys Rep (N Y). 2021 Jul 21;1(1):100004. doi: 10.1016/j.bpr.2021.100004 (PMC8978554; doi:10.1016/j.bpr.2021.100004)
Supplement: Document S1. Supporting materials and methods, Figs. S1–S4, and Tables S1–S3 [file mmc1.pdf]

**Biophysical Reports, Volume *1***

## Supplemental information

# Apolar chemical environments compact unfolded RNAs and can promote folding

**Shamal M. Gunawardhana and Erik D. Holmstrom**

# Apolar chemical environments compact unfolded RNAs and can promote folding

Compaction of RNA in apolar environments (running title)

Shamal M. Gunawardhana[1] and Erik D. Holmstrom[1,2]*

[1]Department of Molecular Biosciences, University of Kansas, Lawrence, Kansas, USA
[2]Department of Chemistry, University of Kansas, Lawrence, Kansas, USA

* To whom correspondence should be addressed. Tel: +1 (785) 864 1712; Email: erik.d.holmstrom@ku.edu

**SUPPLEMENTAL INFORMATION**

Fundamentally, the efficiency (*E*) of Förster resonance energy transfer in a FRET experiment depends on two critical parameters (Eq. S1), both of which have units of distance. The first parameter is the physical nanoscopic distance, *r*, separating the dipoles of the two interacting fluorophores. The second parameter, called the Förster distance ($R_0$), is a numerical quantity (Eq. S2) that specifically characterizes the two interacting fluorophores. The exact value depends on several factors, including: (*i*) the relative spatial orientation of the two interacting dipoles, which is symbolized via $\kappa^2$; (*ii*) the fluorescence quantum yield of the donor fluorophore in the absences of the acceptor fluorophore, which is symbolized via $Q_D$; (*iii*) the overlap integral between the donor emission spectrum and the acceptor absorption spectrum, which is symbolized via *J*; and (*iv*) the refractive index of the medium surrounding the fluorophores, symbolized via $n_D$ (1).

$$E = R_0^6 / (R_0^6 + r^6) \qquad \text{Eq. S1}$$

$$R_0^6 = \frac{9000 \ln(10)\, \kappa^2\, Q_D\, J}{128\, \pi^5 n_D^4\, N_A} \qquad \text{Eq. S2}$$

The numerical value of $R_0$ is on the order of 5 nm, depending on the identity of donor and acceptor fluorophores, and is often assumed to be constant throughout an experiment. In this way, any experimentally observed changes in *E* can be attributed to changes in the inter-fluorophore distance, *r*. However, given that the value of $R_0$ itself depends on several factors, it is possible that experimentally observed changes in *E* may result from changes in $R_0$ as well as *r*.

For example, the refractive index of our binary solvent mixtures is not identical to pure water. However, the values of $n_D$ for these cosolvents (Table S1) do not substantially deviate from that of a purely aqueous solution. From Eq. S2, one can see that the $R_0$ is proportional to $n_D^{-2/3}$. Therefore, small changes to the value of $n_D$ will barely alter the value of $R_0$, which, alone, would not be enough to account for the experimentally observed changes in the value of $\langle E \rangle$.

| Solvent | $n_D$ | $R_0$ $^{(solvent)}$ / $R_0^{(water)}$ |
|---|---|---|
| $H_2O$ | 1.3330 | 1.0000 |
| MeOH * | 1.3287 | 1.0022 |
| EtOH * | 1.3615 | 0.9860 |
| IPA * | 1.3776 | 0.9783 |
| ACN * | 1.3427 | 0.9952 |
| DMK ‡ | 1.3598 | 0.9868 |

**Table S1:** Index of refraction ($n_D$) values at a wavelength that corresponds to the emission of the donor fluorophore (i.e., 570 nm) for the solvents used in this report. The relationship between $R_0$ and $n_D$ (Eq, S2) is used to calculate the expected effect on the Förster distance. The * denotes values taken from (2) and the ‡ denotes values taken from (3).

Additionally, it is well known that organic solvents can also influence the photophysical properties of fluorophores (4), including: (*i*) the fluorescence lifetime, $\tau_{fl}$, and (*ii*) the rotational correlation time, $\tau_{rot}$. Importantly, solvent-dependent changes in $\tau_{fl}$ and $\tau_{rot}$ can alter the value of $R_0$ via $Q_D$ and $\kappa^2$, respectively. To determine how our organic cosolvents influence the values of $\tau_{fl}$ and $\tau_{rot}$ for the donor and acceptor fluorophores additional measurements were performed using interleaved pulsed excitation (5). These experiments were performed on the same microscope system as the alternating continuous-wave excitation experiments described in the main text, however the two dual-mode lasers were set to output ~ 100 µW of short (< 150 ps) pulses at a repetition rate of 20 MHz, which allowed us to lower the temporal resolution of the TCSPC module by more than three orders of magnitude to a value of 16 ps (i.e., $2^4$ ps). Pulsed excitation experiments were conducted on samples containing either water as the only solvent or binary aqueous-organic solvent mixtures at 45% (v/v). In either case, all samples contained 250 mM of KCl, 25 mM HEPES, 12.5 mM NaOH and 0.01% tween 20 (v/v). Fluorescence intensity decay (FID) histograms were constructed from the TCSPC data for the donor-only ($S > 0.9$) and acceptor-only ($S < 0.1$) sub-populations of fluorescently labelled molecules. The FID histograms were then tail-fit using a single decaying exponential function (6) to estimate the fluorescence lifetime of the donor (${}^{Don}\tau_{fl}$) and acceptor (${}^{Acc}\tau_{fl}$) fluorophores (Table S2).

| HP-RNA | | | | |
|---|---|---|---|---|
| **Solvent (v/v)** | $^{Don}\tau_{fl}$ (ns) | $^{Acc}\tau_{fl}$ (ns) | $^{Don}\tau_{rot}$ (ns) | $^{Acc}\tau_{rot}$ (ns) |
| 100% $H_2O$ | 2.72 ± 0.02 | 2.97 ± 0.01 | 0.84 ± 0.22 | 0.62 ± 0.12 |
| 45% MeOH | 2.72 | 3.15 | 0.85 | 0.99 |
| 45% EtOH | 2.68 | 3.21 | 0.92 | 1.18 |
| 45% IPA | 2.67 | 3.23 | 1.04 | 1.35 |
| 45% ACN | 2.62 | 3.41 | 0.70 | 0.64 |
| 45% DMK | 2.66 | 3.26 | 0.86 | 0.84 |
| **TL-RNA** | | | | |
| **Solvent (v/v)** | $^{Don}\tau_{fl}$ (ns) | $^{Acc}\tau_{fl}$ (ns) | $^{Don}\tau_{rot}$ (ns) | $^{Acc}\tau_{rot}$ (ns) |
| 100% $H_2O$ | 2.68 | 2.93 | 0.90 | 0.49 |
| 45% MeOH | 2.66 | 3.14 | 1.14 | 0.53 |
| 45% EtOH | 2.65 | 3.19 | 1.15 | 0.60 |
| 45% IPA | 2.64 | 3.21 | 1.49 | 0.70 |
| 45% ACN | 2.66 | 3.40 | 0.66 | 0.40 |
| 45% DMK | 2.64 | 3.25 | 0.96 | 0.46 |

**Table S2:** Fluorescence lifetime and rotational correlation time of the donor and acceptor fluorophores under various experimental conditions (100 pM RNA, 25 mM HEPES, 12.5 mM NaOH, 250 mM KCl, 0.01% v/v Tween 20, and the specified amount of organic solvent).

In general, the organic cosolvents slightly decreased (< 4%) the fluorescence lifetime of the donor fluorophore, ${}^{Don}\tau_{fl}$. If this slight decrease in lifetime was entirely due to non-radiative processes, then the donor quantum yield ($Q_D$) would also decrease by < 4%. From Eq. S2, one can see that $R_0$ is proportional to the sixth-root of $Q_D$. As such, a 4% decrease in $Q_D$ would have little effect (< 1%) on the $R_0$ and therefore it would not be expected to substantially change the resulting FRET efficiency. For these reasons, any experimentally observed changes in the value of $E$ in apolar conditions are likely due to changes in the inter-fluorophore distance, $r$, rather than $R_0$.

The fluorescence lifetime of the acceptor fluorophore, ${}^{Acc}\tau_{fl}$, is slightly more influenced by the presence of organic cosolvents and increases by as much as 16%. Fortunately, ${}^{Acc}\tau_{fl}$ does not influence the value of $R_0$ and can be accounted for when accurately determining transfer efficiencies via the correction factor associated with the non-identical detection efficiencies of the donor and acceptor fluorophores.

Different solvents can also influence the rotational motion of the fluorophores, leading to changes in the rotational correlation time, $\tau_{rot}$, which can affect the value of $\kappa^2$ associated with $R_0$. Unfortunately, direct measurements of $\kappa^2$ are challenging and therefore a value of $\kappa^2 = 2/3$ is often used, which corresponds to the dynamical average of two fluorophores, each sampling an isotropic distribution of orientations on a time scale that is much faster than $\tau_{fl}$. However, the assumed value of 2/3 may not be appropriate in every situation and the true value of $\kappa^2$ may change with changing experimental conditions.

Fortunately, the rotational correlation time is much easier to experimentally measure and can be used to determine upper and lower limits on the value of $\kappa^2$. To accomplish this, the parallel and perpendicular components of the FID histograms were used to construct fluorescence anisotropy decay (FAD) histograms, which were tail-fit using a single decaying exponential function (7) to estimate the rotational correlation time of the donor (${}^{Don}\tau_{rot}$) and acceptor (${}^{Acc}\tau_{rot}$) fluorophores coupled to the RNA of interest (Table S2). In all cases, the rotational correlation times were always lower in magnitude than the fluorescence lifetimes, suggesting that the two fluorophores were rapidly tumbling in solution. From here, $\tau_{rot}$ is used in conjunction with the Perrin equation (8), $(r_{ss}/r_0) = (1+(\tau_{fl} / \tau_{rot}))^{-1}$, to estimate the depolarization factor for the donor (${}^{Don}d_x$) and acceptor (${}^{Acc}d_x$) fluorophores (9), where $d_x = (r_{ss} / r_0)^{1/2}$, with $r_{ss}$ and $r_0$ representing the steady-state and fundamental fluorescence anisotropies of the fluorophore. Next, the depolarization factors are used to calculate the limiting values of $\kappa^2$ (9). With these numbers in hand, we can then determine minimum and maximum values of $R_0$ relative to a value of $R_0$ assuming $\kappa^2 = 2/3$ (Table S3).

Such an analysis indicates that these solvent-dependent changes in rotational motion of the fluorophore may slightly alter the range of possible values associated with the Förster distances. However, even in the extreme limiting cases the magnitude of this effect is only 10-15%, which would suggest that the observed changes in the experimentally measured transfer efficiency likely arise from changes in inter-fluorophore distance (e.g., biomolecular conformational changes) rather than photophysical changes that would alter the value of $R_0$.

| HP-RNA | | | | |
|---|---|---|---|---|
| **Solvent (v/v)** | ${}^{Don}d_x$ | ${}^{Acc}d_x$ | ${}^{min}R_0/R_0$ | ${}^{max}R_0/R_0$ |
| 100% $H_2O$ | 0.486 | 0.416 | 0.905 | 1.166 |
| 45% MeOH | 0.488 | 0.489 | 0.894 | 1.180 |
| 45% EtOH | 0.506 | 0.518 | 0.887 | 1.188 |
| 45% IPA | 0.529 | 0.543 | 0.880 | 1.197 |
| 45% ACN | 0.459 | 0.398 | 0.911 | 1.157 |
| 45% DMK | 0.494 | 0.453 | 0.899 | 1.174 |
| **TL-RNA** | | | | |
| **Solvent (v/v)** | ${}^{Don}d_x$ | ${}^{Acc}d_x$ | ${}^{min}R_0/R_0$ | ${}^{max}R_0/R_0$ |
| 100% $H_2O$ | 0.501 | 0.379 | 0.908 | 1.161 |
| 45% MeOH | 0.548 | 0.380 | 0.901 | 1.169 |
| 45% EtOH | 0.550 | 0.398 | 0.898 | 1.173 |
| 45% IPA | 0.601 | 0.423 | 0.887 | 1.186 |
| 45% ACN | 0.446 | 0.324 | 0.922 | 1.141 |
| 45% DMK | 0.516 | 0.352 | 0.909 | 1.158 |

**Table S3:** Depolarization factors for the donor and acceptor fluorophores determined using the Perrin Equation and the fluorescence lifetime and rotational correlation time of the respective fluorophores attached to the RNA of interest. Depolarization factors are used to estimate minimum and maximum values for the orientation factor ($\kappa^2$), which are then used to calculate upper and lower bounds for the characteristic Förster distance ($R_0$) in the binary aqueous-organic solvent mixtures (100 pM RNA, 25 mM HEPES, 12.5 mM NaOH, 250 mM KCl, 0.01% v/v Tween 20, and the specified amount of organic solvent).

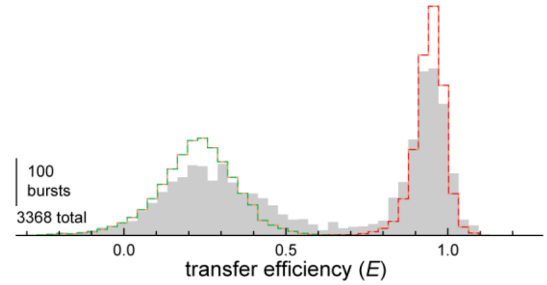


**Figure S1: Near shot noise-limited width of single-molecule transfer efficiency histograms.** Photons from bursts with transfer efficiency values greater than or less than 0.7 were used to generate shot noise-limited histograms for the folded (red) and unfolded sub-populations (green). Such an analysis assumes that true distribution of transfer efficiencies associated with the two sub-populations is infinitely narrow, with values for the mean transfer efficiency, $\langle E \rangle$, and fractional abundance, $\Theta$, of the folded and unfolded sub-populations fixed at those determined via the gaussian fitting procedure described in the main text. The sum of these shot noise-limited histograms (orange) is only slightly narrower than the experimentally observed histograms (gray) recorded under typical solution conditions (100 pM RNA, 25 mM HEPES, 12.5 mM NaOH, 250 mM KCl, 0.01% v/v Tween 20). The excess width associated with the experimental histograms could indicate a small amount of conformational heterogeneity within the folded and unfolded sub-populations.

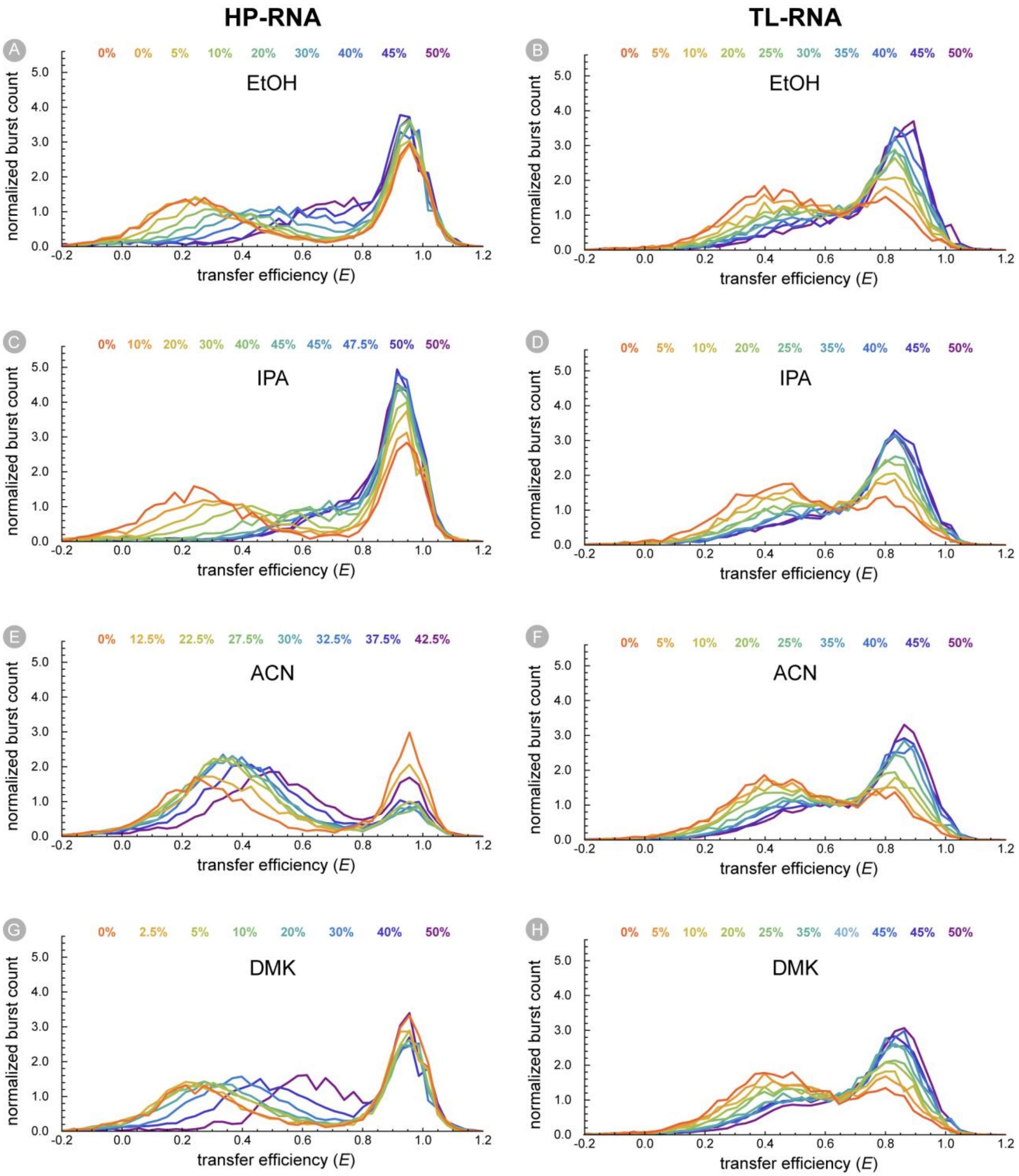


**Figure S2: Transfer efficiency histograms of RNAs in apolar chemical environments.** Transfer efficiency histograms are shown for the HP-RNA (left) and TL-RNA (right) in binary aqueous organic mixtures consisting of water and various amounts of EtOH **(A,B)**, IPA **(C,D)**, ACN **(E,F)**, or DMK **(G,H)**. Solution conditions were as follows: (100 pM RNA, 25 mM HEPES, 12.5 mM NaOH, 250 mM KCl, 0.01% v/v Tween 20, and the specified amount organic solvent) Note: some histograms reflect measurements that were performed under identical experimental conditions.

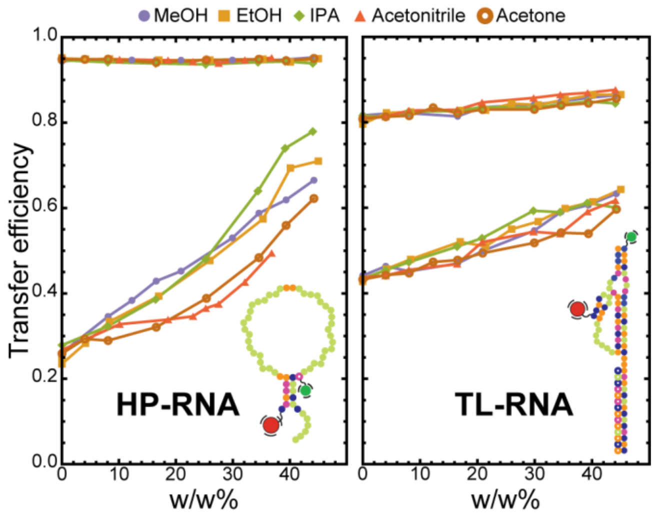


**Figure S3: Polarity of local chemical environment modulates the dimensions of structured RNAs.** This figure displays the information depicted in Figure 4A and 4B of main text. However, the x-axis represents the amount (in weight percent) of organic cosolute in the sample solutions instead of the relative dielectric constant, $\varepsilon_r$, of that cosolvent system (100 pM RNA, 25 mM HEPES, 12.5 mM NaOH, 250 mM KCl, 0.01% v/v Tween 20, and the specified amount of organic solvent).

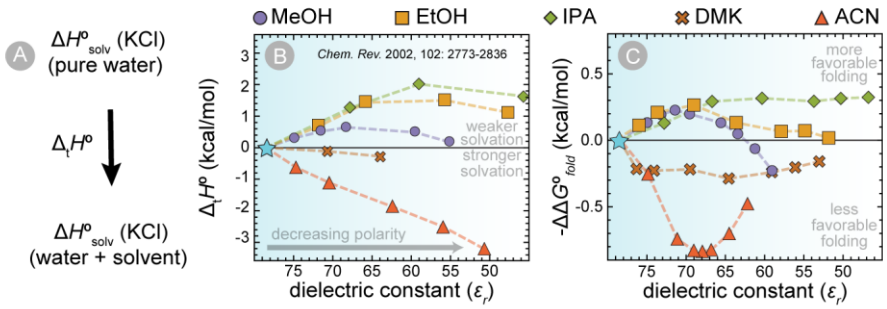


**Figure S4: Solvent Transfer Enthalpies for KCl. (A)** Solvent transfer enthalpy ($\Delta_t H°$) measures the change in the solvation enthalpy ($\Delta H°_{solv}$) that occurs when an electrolyte (e.g., KCl) is transferred from pure water another solvent system (e.g., a binary aqueous organic mixture). It provides an energetic measure of how the solvation of the electrolyte changes, with larger positive values indicating weaker solvation. **(B)** A plot of the reported values (10) of $\Delta_t H°$ associated with transferring KCl from water to the aqueous organic mixtures used in this study. The solvents ACN and DMK strengthen the solvation of KCl, whereas the protic alcohols, IPA EtOH and MeOH, non-monotonically weaken the solvation of KCl. **(C)** Negative values of $\Delta\Delta G°_{fold}$ qualitatively mirror the values of $\Delta_t H°$, suggesting that folding of the HP-RNA becomes more favorable when the solvation of the dominant electrolyte (e.g., KCl) is less enthalpically favorable.


1. Schuler, B. 2013. Single-molecule FRET of protein structure and dynamics - a primer. *J Nanobiotechnology.* 11 Suppl 1:S2, doi: 10.1186/1477-3155-11-S1-S2.

2. Kozma, I. Z., P. Krok, and E. Riedle. 2005. Direct measurement of the group-velocity mismatch and derivation of the refractive-index dispersion for a variety of solvents in the ultraviolet. *J Opt Soc Am B.* 22:1479-1485, doi: Doi 10.1364/Josab.22.001479.

3. Rheims, J., J. Koser, and T. Wriedt. 1997. Refractive-index measurements in the near-IR using an Abbe refractometer. *Meas Sci Technol.* 8:601-605, doi: Doi 10.1088/0957-0233/8/6/003.

4. Anonymous, "Solvent and Environmental Effects" in Principles of Fluorescence Spectroscopy, Lakowicz, J. R., Ed. (Springer US, Boston, MA, 2006), 10.1007/978-0-387-46312-4_6, pp. 205-235.

5. Hendrix, J., and D. C. Lamb. 2013. Pulsed interleaved excitation: principles and applications. *Methods Enzymol.* 518:205-243, doi: 10.1016/B978-0-12-388422-0.00009-1.

6. Anonymous, "Time-Domain Lifetime Measurements" in Principles of Fluorescence Spectroscopy, Lakowicz, J. R., Ed. (Springer US, Boston, MA, 2006), 10.1007/978-0-387-46312-4_4, pp. 97-155.

7. Anonymous, "Time-Dependent Anisotropy Decays" in Principles of Fluorescence Spectroscopy, Lakowicz, J. R., Ed. (Springer US, Boston, MA, 2006), 10.1007/978-0-387-46312-4_11, pp. 383-412.

8. Anonymous, "Fluorescence Anisotropy" in Principles of Fluorescence Spectroscopy, Lakowicz, J. R., Ed. (Springer US, Boston, MA, 2006), 10.1007/978-0-387-46312-4_10, pp. 353-382.

9. Anonymous, "Energy Transfer" in Principles of Fluorescence Spectroscopy, Lakowicz, J. R., Ed. (Springer US, Boston, MA, 2006), 10.1007/978-0-387-46312-4_13, pp. 443-475.

10. Hefter, G., Y. Marcus, and W. E. Waghorne. 2002. Enthalpies and entropies of transfer of electrolytes and ions from water to mixed aqueous organic solvents. *Chem Rev.* 102:2773-2836, doi: 10.1021/cr010031s.
