# Supplementary material for: Apolar chemical environments compact unfolded RNAs and can promote folding
Source: Biophys Rep (N Y). 2021 Jul 21;1(1):100004. doi: 10.1016/j.bpr.2021.100004 (PMC8978554; doi:10.1016/j.bpr.2021.100004)
Supplement: Document S2. Article plus supporting material [file mmc2.pdf]

Biophysical Reports

ARTICLE

Apolar chemical environments compact unfolded RNAs and can promote folding

Shamal M. Gunawardhana[1] and Erik D. Holmstrom[1,2,*]
[1]Department of Molecular Biosciences and [2]Department of Chemistry, University of Kansas, Lawrence, Kansas

ABSTRACT It is well documented that the structure, and thus function, of nucleic acids depends on the chemical environment surrounding them, which often includes potential proteinaceous binding partners. The nonpolar amino acid side chains of these proteins will invariably alter the polarity of the local chemical environment around the nucleic acid. However, we are only beginning to understand how environmental polarity generally influences the structural and energetic properties of RNA folding. Here, we use a series of aqueous-organic cosolvent mixtures to systematically modulate the solvent polarity around two different RNA folding constructs that can form either secondary or tertiary structural elements. Using single-molecule Förster resonance energy transfer spectroscopy to simultaneously monitor the structural and energetic properties of these RNAs, we show that the unfolded conformations of both model RNAs become more compact in apolar environments characterized by dielectric constants less than that of pure water. In the case of tertiary structure formation, this compaction also gives rise to more energetically favorable folding. We propose that these physical changes arise from an enhanced accumulation of counterions in the low dielectric environment surrounding the unfolded RNA.

WHY IT MATTERS The biological functions of noncoding RNAs are often closely related to their ability to form intricate three-dimensional structures. These structures are stabilized by several weak interactions within the RNA and between the RNA, the solvent, and any other solutes in solution. Here, we demonstrate that chemical environments that are less polar than water (and akin to those that an RNA might experience when bound to a protein or in any number of biotechnological applications) generally compact unfolded RNA conformations and, in some cases, can shift conformational equilibria to favor the formation of folded structures. These finding shed light on the physical rules that govern how noncoding RNAs adopt intricate structures essential to all living entities.

INTRODUCTION

Over the years, it has become increasingly apparent that the functions of structured RNAs are intimately related to the many conformations that they adopt in aqueous solutions (1). To better understand the folding of these biological polymers, biophysical and biochemical studies are often conducted in physiologically inspired aqueous solutions that aim to mimic various components (e.g., inorganic or macromolecular) of intracellular environments (2). For example, the role of inorganic counterions (3–6) such as K^+ (7,8) and Mg^{2+} (9,10) in RNA folding has been an active area of research for decades (11,12). Furthermore, the abundance of essential nucleoprotein complexes in biology has led to numerous studies of protein-assisted RNA folding (13), in which certain types of positively charged RNA-binding proteins have been proposed to function as macromolecular counterions that can facilitate conformational transitions in nucleic acids (14,15). From these studies, it is clear that electrostatic contributions can greatly alter the kinetics and energetics of RNA structure formation.

In addition to acting as a counterion, any protein bound to an RNA will also alter the local chemical environment around the RNA, resulting in something that is generally less polar than water (16), which we will refer to as an apolar environment. Because polarity arises from the separation of electrical charges, apolar environments will inevitably alter the electrostatic considerations associated with RNA folding. Unfortunately, a complete understanding of how they influence the structural and energetic aspects of RNA folding has not yet been fully established, with only a handful of

Submitted May 4, 2021, and accepted for publication July 2, 2021.
*Correspondence: erik.d.holmstrom@ku.edu
Editor: Dmitrii Makarov.
https://doi.org/10.1016/j.bpr.2021.100004
© 2021 The Authors.
This is an open access article under the CC BY license (http://creativecommons.org/licenses/by/4.0/).

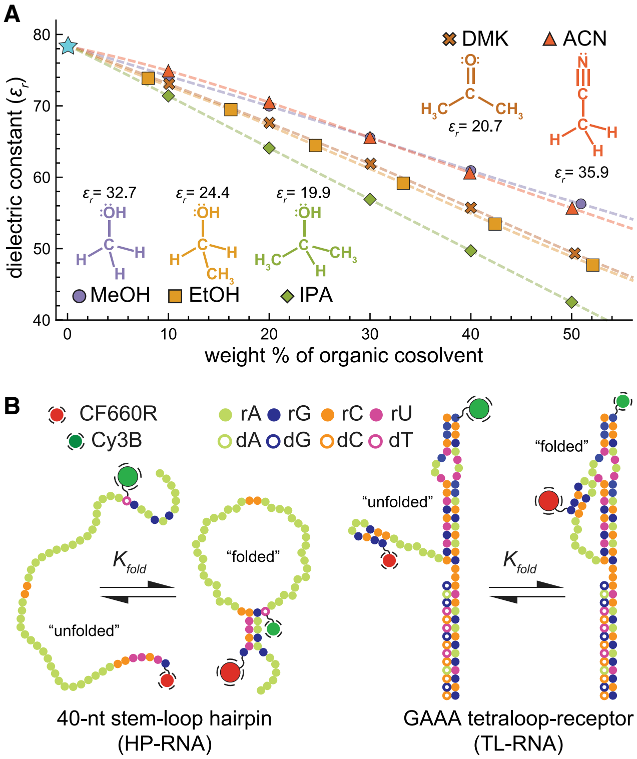

FIGURE 1 Formation of RNA secondary and tertiary structures in apolar environments. The relative dielectric constant (ε_r) is used to quantify solvent polarity. (*A*) Reported values of ε_r for several binary aqueous-organic solvent mixtures at 25°C. Solvent polarity monotonically decreases with increasing amounts of organic solvent. Values for water-MeOH, water-EtOH, and water-DMK were taken from (30). References (31) and (32) were used to derive the dielectric constants of water-IPA and water-ACN mixtures, respectively. (*B*) Color-coded sequence diagrams of the two model RNA constructs used in this study. Folding of the HP-RNA (*left*) results in the formation of five canonical basepairs, which hold together the hairpin secondary structure with a large poly-rA loop. Folding of the TL-RNA (*right*) involves docking the GAAA tetraloop into its cognate receptor. This common tertiary structural motif is held together by several noncanonical interactions (33). For the single-molecule fluorescence studies, both RNAs were modified with Cy3B and CF660R, which function as the FRET donor and acceptor fluorophores, respectively. For both RNAs, the efficiency of energy transfer from the donor to the acceptor is highest in the folded conformation.

ensemble biochemical studies suggesting that they can indeed alter the folding energetics and/or enzymatic activity of RNA (17–25). Not only are the effects of environmental polarity important for a more complete understanding of RNA folding and RNA-protein interactions, but they also have significant implications for biotechnology as well, for which several mainstream applications routinely use solvent systems that are less polar than water, e.g., polymerase chain reaction (26), RNA precipitation (27), phenol-chloroform extraction (28), and reverse-phase high-performance liquid chromatography (29). Therefore, we set out to further investigate the influence of apolar chemical environments on the fundamental principles governing RNA folding using a series of aqueous-organic mixtures (Fig. 1 *A*). Unfortunately, polarity can be a difficult property to quantify, particularly for systems containing multiple cosolvents (34). However, the dielectric constant, or relative permittivity (ε_r), is a bulk property that is closely related to polarity and has been extensively measured for nearly a century (31). Furthermore, many common organic solvents have dielectric constants that are lower than water, and therefore, by mixing water with a miscible cosolvent, we can produce a series of apolar chemical environments for RNA folding studies.

Past efforts to study the structural and energetic properties of RNAs in apolar environments have primarily been carried out using ensemble spectroscopic techniques (18,24,35). Although limited in number, these efforts have started to unveil new insights into the role of polarity in RNA folding. However, the data obtained from these ensemble approaches only report on the average properties of a large population of molecules, making it difficult to identify differential effects on distinct subpopulations of molecules (e.g., folded and unfolded). To avoid these shortcomings, we use single-molecule Förster resonance energy transfer (FRET) to systematically study how different apolar environments influence the structural and energetic aspects of RNA folding using two different model systems, each dissolved in various aqueous-organic cosolvent systems. FRET is a powerful photophysical phenomenon that can be monitored spectroscopically to measure nanometer-scale distances between two neighboring fluorescent probes. When these probes are attached to an RNA of interest, one can use a sensitive fluorescence microscope to measure structural changes within individual molecules (36,37), which make it is possible to obtain information about the conformational and energetics properties of distinct subpopulations associated with heterogeneous molecular ensembles.

The two fluorescently labeled model RNA-folding systems used in this study are 1) a hairpin RNA (HP-RNA) construct (Fig. 1 *B*, *left*) with a 5 basepair stem and a large poly-rA loop (14) and 2) a GAAA tetraloop-tetraloop receptor RNA (TL-RNA) construct (Fig. 1 *B*, *right*) derived from the *Tetrahymena* ribozyme (38). Previous biophysical investigations of these two model RNAs revealed that both display cooperative two-state folding and unfolding behavior during the formation of either secondary (HP-RNA) or tertiary (TL-RNA) structural interactions (14,38). To understand the influence of solvent polarity on the structural and energetic properties of RNA folding, the two FRET-labeled RNAs were each dissolved in different cosolvent systems prepared by mixing water with several different organic solvents: methanol (MeOH); ethanol

(EtOH); isopropyl alcohol (IPA); acetone, which is also known as dimethyl ketone (DMK); and acetonitrile (ACN). The results of these experiments show that apolar chemical environments characterized by dielectric constants lower than that of water invariably compact the unfolded states of these two RNAs, which, in some cases, results in more energetically favorable folding.

MATERIALS AND METHODS

Chemicals and reagents

Doubly distilled water was used to prepare all solutions. KCl, NaOH, Na_2HPO_4, NaH_2PO_4, HEPES, EDTA, MeOH, and IPA were purchased from Sigma-Aldrich (St. Louis, MO). EtOH (190 proof) was purchased from Decon Labs (King of Prussia, PA). DMK and ACN were purchased from EMD Millipore (Burlington, MA) and Thermo Fisher Scientific (Fair Lawn, NJ), respectively. All the chemicals, solvents, and reagents used in this research were of the highest purity grade available at the time.

Preparation of the hairpin RNA construct

A commercially synthesized (Integrated DNA Technologies, Coralville, IA) custom-designed bireactive RNA construct was purchased with the following nucleotide sequence: 5′-/5ThioMC6-D/ GUC UUC CAA AAA AAA AAA AAA AAA AAC CAA AAA AAA AAA AAA AAA AA/iAmMC6T/ GAA GAG AAA AAA-3′ (HP-RNA).

The length of the poly-rA loop and the number of complementary basepairs in the hairpin RNA (HP-RNA) construct were specifically designed to balance the rate constants describing formation and disruption of the 5 basepair duplex such that the folding of this secondary structural element is nearly isoenergetic in solution conditions with near-physiological salt concentrations while also maintaining a conformational change that could be easily resolved with FRET. This bireactive oligonucleotide was dissolved in a buffered aqueous solution to covalently and site-specifically couple the FRET donor and acceptor fluorophores to the RNA. Specifically, a maleimide-functionalized CF660R (acceptor) fluorophore was coupled to the RNA via a 6-carbon thiol-modified phosphate (/5ThioMC6-D/) at the 5′ end of the oligonucleotide and an *N*-hydroxysuccinimide (NHS)-functionalized Cy3B (donor) fluorophore was coupled to the RNA via a 6-carbon amino-modified deoxythymidine (/iAmMC6T/) at nucleotide position 48 (Fig. 1 *B*). The doubly labeled and high-performance liquid chromatography (HPLC)-purified HP-RNA was flash frozen and stored at -70°C until use. A more detailed description of labeling reactions and purification process can be found elsewhere (14).

Preparation of the GAAA tetraloop receptor construct

The three-piece construct was assembled following a previously established protocol (38). The two RNA oligos (ExonanoRNA, Columbus, OH) and one DNA oligo (Integrated DNA Technologies) were purchased as custom-synthesized material with the following sequences: 5′-/Am6/ GGC GAA AGC CAA AAA AAC GUG UCG UCC UAA GUC GGC-3′ (TL-RNA1); 5′-/Am6/ GCC GAU AUG GAC GAC ACG CCC UCA GAC GAG UGCG-3′ (TL-RNA2); 5′-/5Biosg/ CGC ACT CGT CTG AG-3′ (TL-DNA3).

The nucleotide sequences for the two interacting secondary structural elements of the GAAA TL-RNA construct were taken from the *Tetrahymena* ribozyme and linked together with a short single-stranded RNA linker, which controls the effective local concentration of the two species. The length of the linker was chosen such that the resulting folding reaction is nearly isoenergetic in solution conditions with near-physiological salt concentrations while also maintaining a conformational change that could be resolved with FRET. The 6-carbon amino-modified phosphate (/Am6/) at the 5′ ends of both RNA oligos was covalently labeled with either CF660R (TL-RNA1) or Cy3B (TL-RNA2) using NHS-coupling chemistry (Fig. 1 *B*). Briefly, the RNA samples were each dissolved in 0.1 M sodium phosphate buffer at pH 8.0. Approximately, 10 nmol of the RNA was repeatedly diluted to $\sim$500 μL using additional sodium phosphate buffer and concentrated to $\sim$25 μL using 3 kDa MWCO centrifuge filter tube (Millipore) to filter out any small reactive primary amino-groups present in the starting material. The concentrated RNA oligo solutions were each mixed with solutions containing $\sim$10-fold molar excess of either the NHS-functionalized donor or acceptor dyes ($\sim$100 nmol, in $\sim$20 μL dimethyl sulfoxide) and then allowed to incubate for 30 min in dark. After incubation, the reaction mixtures were run through pre-equilibrated (0.1 M sodium phosphate (pH 7.0) with 1 mM EDTA) Zeba spin desalting columns (Thermo Fisher Scientific, Waltham, MA) three consecutive times according to the manufacturer's guidelines to remove excess free dye. The degree of labeling was determined by measuring the absorbance ratio of the purified sample at 260 nm as well as at either 560 nm (Cy3B) or 660 nm (CF660R) using a DS-11 FX + spectrophotometer (DeNovix, Wilmington, DE). The labeled TL-RNA1 and TL-RNA2 oligos were further purified using a reversed-phase C18 column (Dr. Maisch, Ammerbuch-Entringen, Germany) and an HPLC system equipped with a multiwavelength light detector (1260 Infinity II; Agilent Technologies, Santa Clara, CA). After labeling and purification, the TL-RNA construct was assembled by annealing the three oligos (i.e., TL-RNA1, TL-RNA2, and TL-DNA3). The annealing was achieved by incubating 6 nmol aliquots (100 μL each) of the three oligos in 0.1 M sodium phosphate buffer at pH 7.0 with 1 mM EDTA for 2 h at room temperature. Annealed TL-RNA construct was flash frozen in liquid nitrogen and stored at -70°C until use. A biotin linker (/5Biosg/) was included in the design of the TL-RNA to enable future single-molecule FRET experiments in which the RNA will be immobilized to the surface of a glass coverslip.

Sample preparation

Measurements were performed in 15-well microscope slides (Ibidi, Planegg, Germany) containing 40 μL of the RNA sample solution, which was prepared using an appropriate volume of concentrated RNA stock solution diluted into the measurement buffer to achieve a final RNA concentration of $\sim$100 pM. The resulting RNA sample solution also contains the following components: 25 mM HEPES, 12.5 mM NaOH, 250 mM KCl, 0.01% v/v Tween 20, and a specified amount of organic solvent. HEPES and NaOH were used to buffer the solution pH. KCl was added to ensure that the folded and unfolded states of the two model RNA constructs were approximately equally populated in the absence of organic solvents and thus that the folding reactions were nearly isoenergetic. Tween 20 was added to prevent adsorption of the RNA to the hydrophobic surfaces of the 15-well microscope slides. Organic solvents were used to lower the dielectric constant of the cosolvent system. For each measurement, the final value of ε_r was interpolated from published data on binary aqueous-organic solvent mixtures (30–32).

Single-molecule FRET spectroscopy with alternating continuous-wave excitation

All single-molecule measurements were performed using a slightly modified MicroTime200 system (PicoQuant, Berlin, Germany) consisting of an inverted confocal fluorescence microscope equipped with two excitation sources and four detectors (Fig. 2 *A*). Two dual-mode

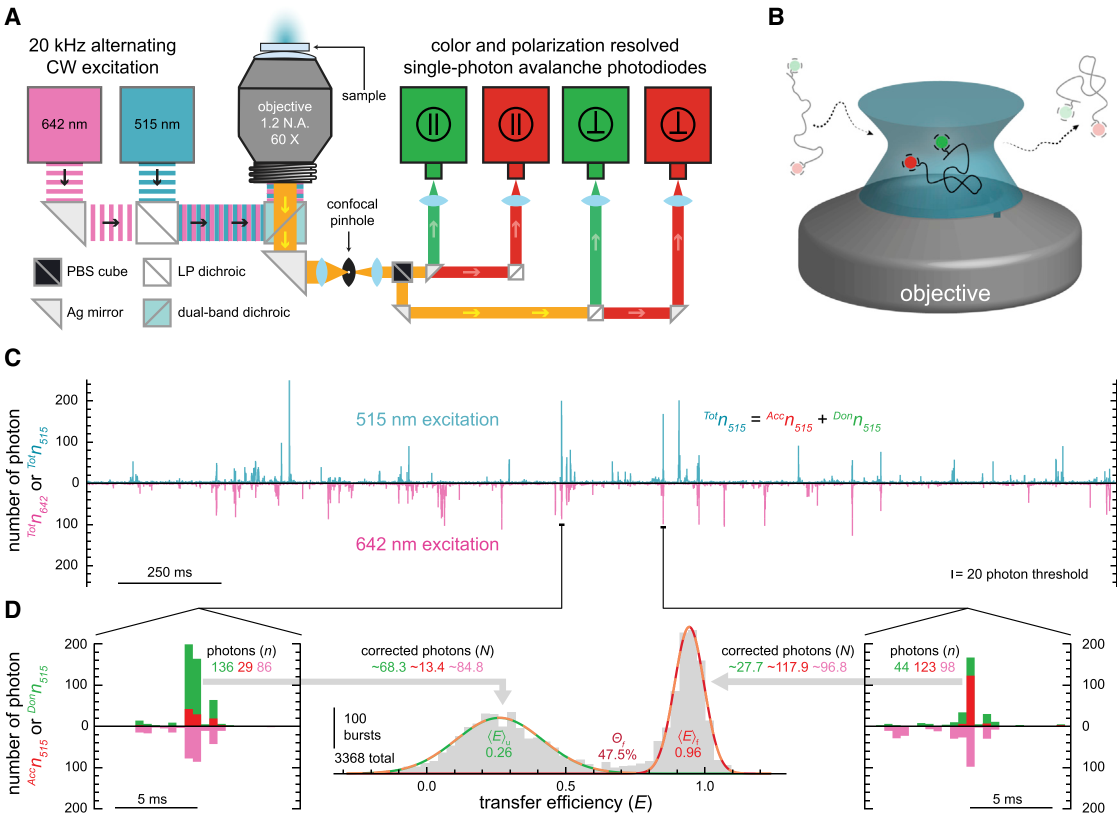

FIGURE 2 Experimental design and analysis. (*A*) Schematic overview of a single-molecule confocal fluorescence microscope. The alternating (20 kHz) output from two diode lasers (515 and 642 nm) is directed into the back aperture of the water immersion objective using a dual-band dichroic mirror resulting in a diffraction-limited focal spot. The stokes-shifted fluorescence emitted from the sample is collected by the same objective and directed through a 100 μm pinhole to remove out-of-plane light. A polarizing beam splitter (PBS) cube and a set of long-pass (LP) dichroic mirrors spatially separates the emission into four photon streams based on polarization and color. The emitted photons in each of the four streams are detected by four separate single-photon avalanche photodiodes. (*B*) Cartoon depicting the confocal volume of an inverted confocal microscope (not to scale). The donor- and acceptor-labeled RNA molecules that stochastically diffuse through the confocal volume get excited by the focused laser beams producing a burst of photons. (*C*) Representative 2.5 s segment of a 900 s fluorescence time trace with 500 μs time bins depicting numerous bursts of photons resulting from alternating 515 and 642 nm excitation of the FRET-labeled RNAs (100 pM RNA, 25 mM HEPES, 12.5 mM NaOH, 250 mM KCl, 0.01% v/v Tween 20). (*D*) Insets on the left and right are representative 12.5 ms segments of the data in (*C*), each depicting a burst of photons. Transfer efficiency (E) and stoichiometry (S) values are calculated for each burst of photons recorded during the measurement using Eq. 1 and Eq. 2, respectively. The values of E arising from those bursts with more than 50 total corrected photons emitted by FRET-labeled molecules (i.e., $0.25 < S < 0.75$) are then compiled into a transfer efficiency histogram. Histograms are fitted to a sum of two Gaussian functions to quantify the mean transfer efficiency, $\langle E \rangle$, and fractional abundance, Θ, of the folded and unfolded subpopulations, with typical experimental uncertainties of $\langle E \rangle \pm 0.02$ and $\Theta \pm 0.03$.

diode lasers (QuixX 515-80PS and QuixX 642-140PS; Omicron, Rodgau, Germany) operating in the continuous-wave mode are used to produce ~100 μW of vertically polarized light at wavelengths of 515 and 642 nm to excite the donor and acceptor fluorophores, respectively. Alternating excitation (39) of the two fluorophores is carried out at 20 kHz using a signal generator (577-4C; Berkeley Nucleonics, San Rafael, CA) to modulate the output of the two lasers. After being reflected off a dual-band dichroic mirror (ZT532/640rpc; Chroma, Bellows Falls, VT), the laser light is then focused to a diffraction-limited spot within the sample using a 60× magnification 1.2 NA water immersion microscope objective (UPlanSAPO; Olympus, Tokyo, Japan). The fluorescence emitted from the sample is collected using the same objective in an epifluorescence configuration and directed toward the detection unit, where it first passes through a 100-μm-diameter confocal pinhole. Then, the emission is spatially separated via a polarizing beam splitter (BS Cube Polarizer; PicoQuant), resulting in two streams of linearly polarized fluorescence: one parallel to the polarization of the laser light, the other perpendicular. The two streams are then each split again using a long-pass dichroic mirror (635LPXR; Chroma) to spectrally resolve donor and acceptor emission, resulting in a total of four separate detection streams. Band-pass filters in the appropriate detection streams reject any fluorescence not associated with the donor (582/64 BrightLine; Semrock, New York, NY) and acceptor (690/70 BrightLine; Semrock) fluorophores. Each stream of fluorescence terminates at a single-photon avalanche photodiode-based detector (SPCM-AQRH-14-TR; Excelitas Technologies, Waltham, MA), where the arrival times of individual photons are recorded by a time-correlated single-photon counting module (HydraHarp 400; PicoQuant) with the temporal resolution set to 16,384 (2^{14}) ps. The duration of each measurement was 900 s.

Data analysis

Under our experimental conditions, the confocal volume is most often devoid of fluorescent molecules, with an average occupancy

of less than 0.01 molecules. Nevertheless, individual fluorescently labeled molecules will inevitably diffuse through the confocal volume, where they will be excited by the two alternating lasers, resulting in a transient burst of fluorescent photons (Fig. 2 *B*). The detection records of the photons emitted from these freely diffusing fluorescently labeled RNA molecules were analyzed using Mathematica 12.0 (Wolfram Alpha) in conjunction with Fretica, a C++ based MATHLINK module for the analysis of time-correlated single-photon-counting single-molecule FRET data (40). The data analysis workflow employed during this research is as follows: first, time-gating was used to determine which laser (either 515 or 642 nm) was active for every detected photon in each of the four streams. Then, photons were assigned to 500 μs time bins based on their absolute arrival time (Fig. 2 *C*). Time bins with a total photon count rate (${}^{Tot}n = {}^{1}n_{515} + {}^{1}n_{642} + {}^{2}n_{515} + {}^{2}n_{642} + {}^{3}n_{515} + {}^{3}n_{642} + {}^{4}n_{515} + {}^{4}n_{642}$) of less than 20 photons per bin were used to calculate the average background photon count rate for each of the four streams during either 515 or 642 nm excitation. Then, corrected photon count rates (N) were determined for all time bins by accounting for background, spectral cross talk, direct excitation of the acceptor, and nonidentical excitation and detection efficiencies of the donor and acceptor fluorophores. Those time bins with a corrected total photon count rate (${}^{Tot}N = {}^{1}N_{515} + {}^{1}N_{642} + {}^{2}N_{515} + {}^{2}N_{642} + {}^{3}N_{515} + {}^{3}N_{642} + {}^{4}N_{515} + {}^{4}N_{642}$) of more than 20 photons per bin were considered bursts of fluorescence arising from single molecules diffusing through the confocal volume (41). Corrected photon count rates associated with the acceptor and donor fluorophores (${}^{Acc}N = {}^{1}N + {}^{3}N$ and ${}^{Don}N = {}^{2}N + {}^{4}N$, where ${}^{Tot}N = {}^{Acc}N + {}^{Don}N$) during 515 and 642 nm excitation were used to calculate values for both the FRET efficiency (E) and fluorescence stoichiometry (S) using Eq. 1 and Eq. 2, respectively (Fig. 2 *D*).

$$E = {}^{Acc}N_{515} / \left({}^{Acc}N_{515} + {}^{Don}N_{515} \right). \tag{1}$$

$$S = {}^{Tot}N_{515} / \left({}^{Tot}N_{642} + {}^{Tot}N_{515} \right). \tag{2}$$

The fluorescence stoichiometry (S) of a burst arising from a molecule containing only donor or acceptor fluorophores will yield values near $S = 1$ and $S = 0$, respectively. Therefore, values of fluorescence stoichiometry are used to restrict our analysis and interpretation of FRET efficiencies to only those bursts arising from molecules containing active donor and acceptor fluorophores (i.e., $0.25 < S < 0.75$). This allows us to effectively filter out unwanted contributions from any potential donor-only or acceptor-only molecules that, for example, may not have been removed during the HPLC purification of the RNA constructs.

The FRET efficiency (E) values from bursts with ${}^{Tot}N > 50$ were then compiled into histograms (Fig. 2 *D*), in which the widths of the resulting distributions were largely limited by shot noise (Fig. S1). Histograms were then fitted using Gaussian distributions to determine the mean FRET efficiency, $\langle E \rangle$, and fractional abundance, Θ, of the folded and unfolded subpopulations. Based on several repeated measurements under identical conditions, typical experimental uncertainties associated with $\langle E \rangle$ and Θ are ± 0.02 and ± 0.03, respectively. The fractional abundance of each subpopulation was used to calculate the equilibrium constant for folding ($K_{fold} = \Theta_f / \Theta_u$) and thus the standard state Gibbs free energy difference ($\Delta G^{\circ}_{fold} = -RT \ln K_{fold}$, where R is the gas constant) between the two subpopulations at $T = 294.2$ K. For the ease of data interpretation, all ΔG°_{fold} values in apolar solvent conditions were referenced to aqueous conditions, i.e., $\Delta\Delta G^{\circ}_{fold} = \Delta G^{\circ}_{fold}$ (mixture) $- \Delta G^{\circ}_{fold}$ (H_2O).

RESULTS

Although the importance of electrostatics in RNA folding has been extensively studied over the past 50 years, we are only beginning to understand how the polarity of the local chemical environment around the RNA influences the structural and energetic aspects of folding. As such, the main objective of this study is to determine how local chemical environments that are less polar than water, and perhaps akin to those experienced by an RNA at the surface of a protein (16), influence the folding of structured nucleic acids. To accomplish this task, we chose to use binary mixtures of water and various organic solvents to systematically adjust the polarity of buffered solutions containing either secondary or tertiary structural elements capable of folding into well-defined structures. Our findings indicate that the apolar environments provided by these cosolvent systems profoundly impact the structural properties of RNAs as well as the energetics of folding.

Dielectric constant of binary aqueous-organic solvent mixtures

Several different scales have been introduced to characterize the polarity of pure solvents. Empirical scales for solvent polarity are often based on a variety of spectroscopic, equilibrium, or kinetic measurements of the solvent or solutes dissolved within it (34). Multiparameter approaches have also been developed that are based on other measurable physical properties of the solvent, such as the refractive index or dielectric constant (34). Despite these efforts, solvent polarity remains difficult to quantify, particularly for miscible aqueous-organic mixtures (34). Therefore, we simply chose to use literature-reported values of the relative dielectric constant (or permittivity), ε_r, to quantify the changing polarity of these cosolvent systems (Fig. 1 *A*). In this study, the structural and energetic aspects of RNA folding were studied in the presence of different binary mixtures of water and various amounts of MeOH, EtOH, IPA, ACN, and DMK. Because the dielectric constants of these pure organic solvents are lower than that of pure water, increasing amounts of organic solvent produce mixtures with decreasing values of ε_r (Fig. 1 *A*).

Two model RNA constructs

The donor and acceptor fluorophores of the HP-RNA construct are separated in primary structure by a long sequence of adenosines (see Materials and methods). Importantly, the five complementary nucleotides on either side of this sequence allow the two ends of the

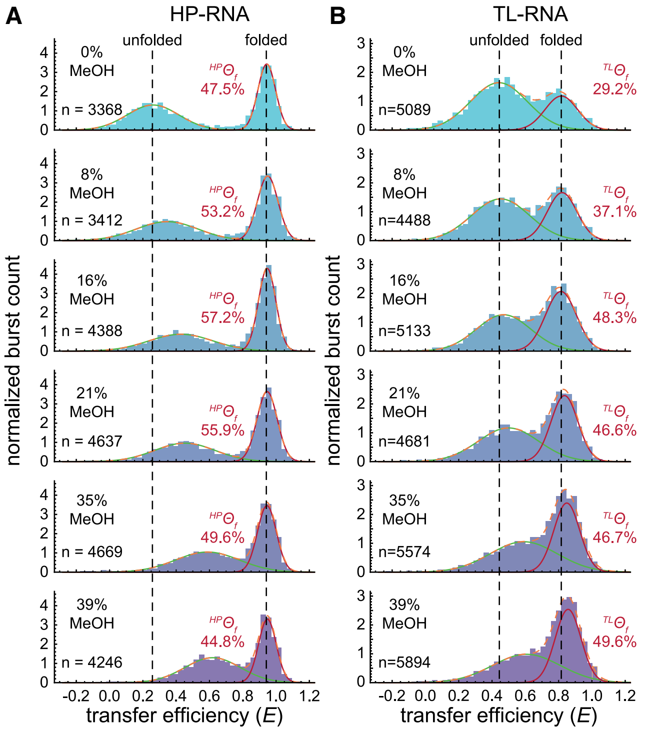

FIGURE 3 Effect of MeOH on RNA folding. Single-molecule transfer efficiency histograms for the HP-RNA (*A*) and TL-RNA (*B*) constructs in various mixtures of H_2O-MeOH are shown. Vertical dashed lines show how the high- and low-transfer-efficiency subpopulations change relative to the pure aqueous conditions (*top*). For the HP-RNA construct, the addition of MeOH as a cosolvent greatly increases the mean transfer efficiency of the unfolded subpopulation while slightly modulating the fractional abundance of the folded subpopulation. For the TL-RNA construct, MeOH increases the mean transfer efficiency of both subpopulations in addition to systematically increasing the fractional abundance of the folded subpopulation (100 pM RNA, 25 mM HEPES, 12.5 mM NaOH, 250 mM KCl, 0.01% v/v Tween 20, and the specified amount MeOH).

oligonucleotide to temporarily basepair with each other (14), resulting in the formation of a short, duplex-like, secondary structural element (Fig. 1 *B*). The HP-RNA molecules that adopt more expanded and unfolded conformations will have lower transfer efficiency values than the more compact folded conformations. Similarly, the fluorophores of the TL-RNA construct are located near the GAAA tetraloop and its cognate receptor. When the tetraloop docks into its receptor, the resulting tertiary structure is temporarily held in place by multiple noncanonical hydrogen-bonding and base-stacking interactions (33). In this way, folding of the TL-RNA brings the two fluorophores closer together, giving rise to molecules with higher transfer efficiencies than their unfolded counterparts (38). For both RNAs, structure formation is governed by the molecular forces they experience in solution, including Coulombic repulsions between nucleotide phosphates, van der Waals interactions, hydrogen bonds, and steric clashes. Any alterations to the above factors, including those brought about by the polarity of the local chemical environments, can affect the structure and relative energetic stability of the folded and unfolded conformations.

RNA folding in an aqueous solvent

In single-molecule FRET measurements under standard aqueous conditions, both RNA constructs adopt two distinct conformations, as indicated by the bimodal distributions observed in transfer efficiency histograms (Fig. 3, *A* and *B*, *top*). The mean transfer efficiency and fractional abundance of each subpopulation were quantified by fitting the distributions to a sum of two normal distributions (see Materials and methods). For the HP-RNA (Fig. 3 *A*, *top*), the high-transfer-efficiency folded subpopulation has a mean value of $^{HP}\langle E\rangle_f \approx 0.95$ and the low-transfer-efficiency unfolded subpopulation has a mean value of $^{HP}\langle E\rangle_u \approx 0.25$. Under these conditions, the fractional abundance of the folded conformation is $^{HP}\Theta_f = 47.5\%$. The folded and unfolded conformations of the TL-RNA construct (Fig. 3 *B*, *top*) are located at slightly more intermediate transfer efficiencies with $^{TL}\langle E\rangle_f \approx 0.8$ and $^{TL}\langle E\rangle_u \approx 0.4$, respectively. Here, the fractional abundance of the folded conformation of the TL-RNA is $^{TL}\Theta_f = 29.2\%$.

Importantly, the fractional abundance of the folded and unfolded conformations can then be used to estimate the equilibrium constant and thus the standard state Gibbs free energy change associated with these two conformational equilibria. For the two RNA constructs under standard aqueous conditions, we get $^{HP}\Delta G^\circ{}_{fold} = 0.056$ kcal mol^{-1} and $^{TL}\Delta G^\circ{}_{fold} = 0.525$ kcal mol^{-1} for the HP-RNA and TL-RNA constructs, respectively. These free energy values under our standard aqueous conditions will serve as a reference point for additional measurements conducted in binary aqueous-organic solvent mixtures.

RNA folding in apolar environments

To study the structural and energetic properties of RNA folding in apolar chemical environments, we carried out similar measurements in binary aqueous-organic solvent mixtures consisting of increasing amounts of MeOH (Fig. 3). From these histograms, one can see how the mean transfer efficiency, $\langle E\rangle$, and fractional abundance, Θ, of the two distinct subpopulations change as the RNAs are exposed to increasingly apolar conditions. To further elucidate the structural and energetic aspects of RNA folding in apolar chemical environments, additional single-molecule FRET experiments were conducted using EtOH and IPA as additional protic solvents, as well as ACN and DMK as aprotic solvents (Fig. S2).

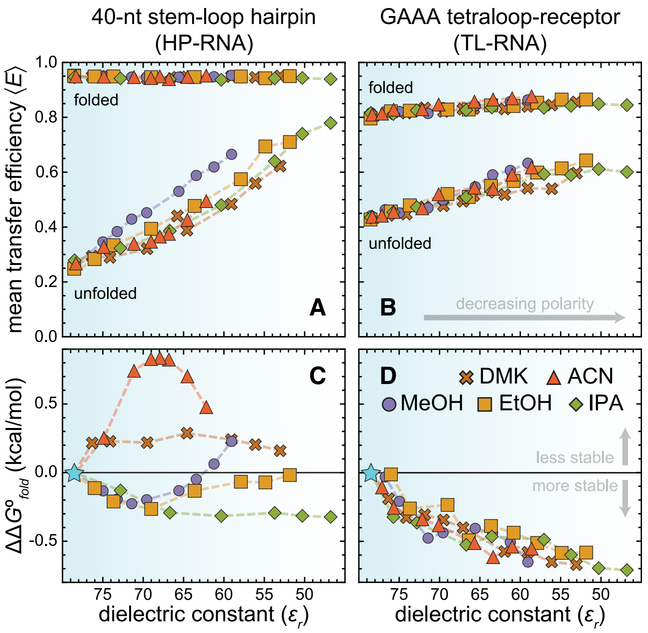

FIGURE 4 Apolar chemical environments compact unfolded RNAs. The mean transfer efficiencies of the folded and unfolded subpopulations of the (*A*) HP-RNA and (*B*) TL-RNA constructs plotted against the relative dielectric constant, ε_r, of various binary aqueous-organic solvent mixtures. Apolar conditions, characterized by values of ε_r that are less than pure water ($\approx$ 78.5), strongly affect the mean transfer efficiency of the unfolded conformations of both RNAs, with almost no dependence on cosolvent identity. Notably, the mean transfer efficiency of folded HP-RNA, $^{HP}\langle E\rangle_f$, and the folded TL-RNA, $^{TL}\langle E\rangle_f$, were much less sensitive to changes in ε_r. The differential free energy change for RNA folding ($\Delta\Delta G^{\circ}_{fold}$) in apolar environments is shown for two RNA constructs designed to probe the formation of (*C*) secondary structure (HP-RNA) and (*D*) tertiary structure (TL-RNA). Based on our definition of $\Delta\Delta G^{\circ}_{fold}$ (see Materials and methods), negative values represent conditions that stabilize the folded conformation (100 pM RNA, 25 mM HEPES, 12.5 mM NaOH, 250 mM KCl, 0.01% v/v Tween 20, and an amount of organic solvent to achieve the specified value of ε_r. The corresponding weight percentages for each measurement are shown in Fig. S3).

Apolar chemical environments compact unfolded RNAs

Upon inspection of the MeOH data sets (Fig. 3), one can clearly see that increasingly apolar environments, containing higher amounts of organic cosolvent, tend to increase the mean transfer efficiency of the unfolded subpopulations of both the HP-RNA and TL-RNA constructs. This trend can be quantified and visualized in plots of $\langle E\rangle$ vs. ε_r (Fig. 4, *A* and *B*), where ε_r systematically decreases as the amount of organic solvent increases. The same general trend is also apparent when using any of the other four organic cosolvents, i.e., more apolar environments, characterized by lower values of ε_r, consistently yield higher values of $^{HP}\langle E\rangle_u$ and $^{TL}\langle E\rangle_u$.

The overall magnitude of the increase is largely independent of cosolvent identity, suggesting that other, nondielectric properties of these aqueous-organic mixtures (e.g., index of refraction) are not responsible for the observed trends (Table S1). Additionally, minor solvent-dependent changes in the photophysical properties of the fluorophores (e.g., fluorescence lifetime) cannot account for the observed changes in mean transfer efficiency (Tables S2 and S3). Therefore, this systematic shift to higher mean transfer efficiencies suggests that the unfolded conformations associated with the HP-RNA (Fig. 4 *A*) and TL-RNA (Fig. 4 *B*) constructs become more compact in apolar environments.

Interestingly, the mean transfer efficiency of the folded conformation of the HP-RNA was largely insensitive to the polarity of the local chemical environment. This finding likely suggests that the geometry and orientation of the canonical basepairing interactions responsible for the formation of RNA secondary structure are not heavily influenced by the polarity of the local chemical environment. Presumably, this is because the bases are sequestered within the interior of the resulting duplex, where the polarity of the local chemical environment is dominated by the dielectric properties of the nucleic acid rather than the solvent. However, our quantitative analysis does reveal a subtle increase in the mean transfer efficiency associated with the folded conformation of the TL-RNA construct in aqueous-organic mixtures (Fig. 4 *B*). Although the change in transfer efficiency is quite small, it persists across all cosolvents, and therefore this finding may be indicative of a slight molecular compaction and/or conformational rearrangement that occurs when RNA tertiary structures are exposed to apolar conditions.

Apolar chemical environments can make folding more energetically favorable

One of the many advantages of single-molecule FRET over conventional ensemble methods is its ability to study individual subpopulations (e.g., folded and unfolded) within a heterogeneous sample. The transfer efficiency histograms for both the HP-RNA and TL-RNA clearly exhibit at least two easily distinguishable subpopulations across a wide range of solution conditions (Fig. 3). Using our Gaussian fitting analysis, we were able to calculate the fractional abundance, Θ, of the folded and unfolded species in each of the measurements, which reflects the relative concentrations of the two subpopulations in solution.

The direct relationship between the folding equilibrium constant ($K_{fold} = \Theta_f/\Theta_u$) and the Gibbs free energy change associated with folding ($\Delta G^{\circ}_{fold} = -RT\ \ln K_{fold}$) allows us to use our single-molecule FRET data to determine how the polarity of the local chemical environment alters the energetics of RNA folding. To accomplish this, we calculate the difference between ΔG°_{fold} in the binary aqueous-organic solvent mixtures

and pure water, where $\Delta\Delta G^\circ_{fold} = \Delta G^\circ_{fold}$ (mixture) $-$ ΔG°_{fold} (H_2O). As summarized in the plots of $\Delta\Delta G^\circ_{fold}$ vs. ε_r (Fig. 4, *C* and *D*), folding free energies associated with the two RNA constructs responded quite differently to increasing amounts of the various organic cosolvents. Indeed, previous investigations of nucleic acid folding in aqueous-organic mixtures have also reported differential effects on the energetics of secondary and tertiary structure formation (19,21). Here, the values of $\Delta\Delta G^\circ_{fold}$ for TL-RNA systematically increase under apolar experimental conditions with lower values of ε_r (Fig. 4 *D*), suggesting that the energetic stability of this tertiary interaction is primarily influenced by the changing polarity. In contrast, the values of $\Delta\Delta G^\circ_{fold}$ for the HP-RNA vary nonmonotonically with increasing organic composition and are highly dependent on the identity of the cosolvent (Fig. 4 *C*), clearly indicating that cosolvent polarity is not the only physicochemical properties of these cosolvents that influences the folding energetics of this RNA.

Upon further inspection, our data provide a potential explanation for the highly variable folding energetics associated with the HP-RNA (Fig. 4 *C*). First, at low to intermediate concentrations, the protic cosolvents tend to be stabilizing, whereas the aprotic cosolvents tend to be destabilizing. Second, the changes in folding energetics for the HP-RNA correlate quite well with the cosolvents ability to act as a hydrogen-bond acceptor (HBA), as quantified via the β_1 scale (42). Specifically, folding becomes slightly more favorable in solutions where the cosolvents are relatively strong HBAs (i.e., ${}^{IPA}\beta_1 = 0.68$; ${}^{EtOH}\beta_1 = 0.62$; ${}^{MeOH}\beta_1 = 0.54$), whereas folding becomes slightly more unfavorable in solutions where the cosolvents are less effective HBAs (i.e., ${}^{DMK}\beta_1 = 0.49$; ${}^{ACN}\beta_1 = 0.37$). Together, these observations seem to indicate that these aqueous-organic mixtures not only alter the polarity of the solution, but they also alter hydrogen-bonding interactions within or between the solvent and the solutes (e.g., RNA and ions) and that these alterations noticeably influence the energetics associated with HP-RNA folding.

Furthermore, the cosolvent identity and concentration-dependence of $-\Delta\Delta G^\circ_{fold}$ for the HP-RNA (Fig. 4 *C*) mirrors reported values (43) for the change in solvation entropy (ΔH°_{solv}) associated with transferring KCl from pure water to a binary aqueous-organic solvent mixture (Fig. S4). The strong relationship between these two thermodynamic properties suggests that the folding energetics observed for the HP-RNA are not dominated by the changing polarity (as observed with the TL-RNA), but rather that they are dominated by the changing energetics of counterion solvation. More specifically, folding of the HP-RNA becomes more favorable when solvation of the dominant salt (e.g., KCl) is less enthalpically favorable relative to pure water, making it easier for the RNA to displace the solvent and engage in direct electrostatic interactions with the ion. Because this behavior was not apparent for both RNAs, it is likely that any potassium ions taken up during folding of the HP-RNA are partially desolvated and directly coordinated by the RNA, whereas the ions taken up during the folding of the TL-RNA (8) are likely to remain fully solvated.

RNA folding in salty environments

The above results show that apolar chemical environments compact the unfolded structural ensembles of both RNAs and, in the case of tertiary structure formation, shift the conformational equilibrium to favor folding. These observations are surprisingly similar to the effect that monovalent cations have on the structural and energetic properties of RNA folding (14,44), perhaps because both can profoundly alter the electrostatics of RNA folding. To more robustly assess these similarities, measurements were conducted on the HP-RNA and TL-RNA in purely aqueous solutions over a wide range of KCl concentrations (Fig. 5). As expected, the unfolded conformations of the two model RNAs also became more compact upon addition of KCl. Furthermore, this compaction was also accompanied by a stabilization of the folded conformations associated with both constructs.

DISCUSSION

It is generally accepted that the molecular structures arising from RNA folding are held in place by hydrogen bonds, the stacking of the aromatic bases, and electrostatic interaction between the negatively charged phosphates and any counterions present in solution (45,46). Over the years, numerous research endeavors have been conducted with the goal of providing a fundamental understanding of these inter- and intramolecular interactions. However, we are only beginning to understand how the polarity of the local chemical environment influences the structural and energetic aspect of the folding process.

In a recent study, Nordén and co-workers observed local breathing and unstacking of double-stranded DNA in "semihydrophobic" solutions containing water and various amounts of ethylene glycol ethers such as diglyme and PEG-400 (47). Using ensemble spectroscopic techniques, Nakano and Sugimoto have also suggested that aqueous-organic solvent mixtures can alter the structural stability and catalytic activity of various nucleic acids (17–25) and generally attribute these effects to decreases in both the dielectric constant and thermodynamic activity of water. However, the insights gleaned from these studies are not strictly limited to a fundamental understanding of the physical

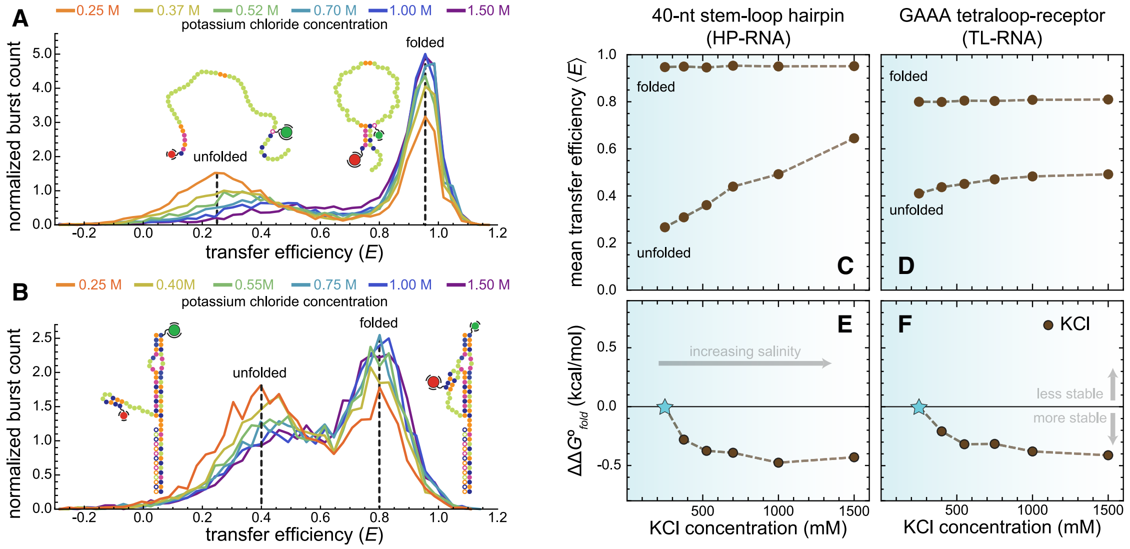

FIGURE 5 Effect of KCl concentration on the conformational dimensions and folding equilibria of RNA secondary and tertiary structure formation. Transfer efficiency histograms show compaction of the unfolded conformations of the (*A*) HP-RNA and (*B*) TL-RNA at elevated KCl concentrations. Conversely, the concentration of KCl has almost no effect on the mean transfer efficiency associated with folded conformations of the HP-RNA and TL-RNA, as shown by the dashed line at $^{HP}\langle E\rangle_f \approx 0.95$ and $^{TL}\langle E\rangle_f \approx 0.8$, respectively. However, in both cases, the two RNAs tend to favor the folded conformation at high concentrations of KCl (100 pM RNA, 25 mM HEPES, 12.5 mM NaOH, 0.01% Tween 20, and, unlike the previous experiments, no organic solvent but instead variable concentrations of KCl). Mean transfer efficiencies of the folded and unfolded subpopulations of the (*C*) HP-RNA and (*D*) TL-RNA constructs are plotted against KCl concentration. The differential free energy change for folding ($\Delta\Delta G^{\circ}_{fold}$), relative to 250 mM KCl, associated with the HP-RNA and (*E*) TL-RNA (*F*) is plotted against KCl concentration.

principles governing RNA folding. For example, the cytosolic environment is also thought to be slightly apolar, and therefore, aqueous-organic solvent mixtures have also been used to mimic the cellular milieu (19). The effects of solvent polarity on RNA folding may also shed light on the molecular function of many important macromolecular machines assembled from both protein and nucleic acid subunits because the surfaces of these protein subunits are substantially less polar than the surrounding aqueous environment (16,20).

In this study, we explored the effect of solvent polarity on both the structural and energetic properties of RNA secondary and tertiary structure formation using single-molecule fluorescence techniques. Importantly, the use of single-molecule approaches allows us to simultaneously monitor the structural and energetic properties of RNAs using quantitative experimental observables (i.e., the mean transfer efficiency $\langle E\rangle$ and Θ, the fractional abundance) associated with distinct subpopulations (e.g., folded and unfolded) within heterogeneous molecular ensembles. We chose to systematically alter solvent polarity, quantified via its dielectric constant, by mixing increasing weight percentages of five different organic solvents with water (Fig. 3 *A*). Our results show a clear compaction of unfolded RNAs in increasingly apolar environments with dielectric constants less than that of pure water, regardless of which solvents are used. The overall extent of compaction, reflected by an increase in the $\langle E\rangle$, scales with the decreasing dielectric constant of the binary aqueous-organic solvent mixtures (Fig. 4, *A* and *B*). Importantly, a similar degree of compaction can also be achieved by simply increasing the concentration of monovalent cations in a purely aqueous solution (Fig. 5). How can we explain these observations?

As described by Coulomb's law (Eq. 3), the magnitude of the electrostatic force, F, between two species separated by a distance, r, with net charges, q_1 and q_2, is inversely proportional to the relative dielectric constant or permittivity, ε_r, of the surrounding medium.

$$F \propto \frac{1}{4\pi\varepsilon_r}\frac{q_1 q_2}{r^2}. \tag{3}$$

Thus, the attractive force between an RNA molecule and any neighboring counterions in solution will increase in apolar environments where the value of ε_r is less than that of pure water. The enhanced Coulombic interaction between 1) the electrostatic potential generated by negatively charged phosphates and 2) nearby solvated cations will increase the total positive charge density in the ion atmosphere (48) surrounding the RNA. This uptake of counterions from the bulk will then lead to more effective screening of the intramolecular electrostatic repulsion between phosphate groups relative to a purely aqueous solvent, allowing the unfolded RNA to more readily sample compact structures. Indeed, our experimental observations in

apolar conditions are remarkably consistent with such a prediction.

Furthermore, the compaction of these unfolded RNAs increases the effective local concentration of the nucleotides involved in the formation of higher-order structures. Compaction may also bias the unfolded ensemble to mimic the compact transition states commonly observed in RNA folding experiments (49–51). In both cases, the net result would be an RNA that is primed to fold, which would increase the folding rate constant and lead to more energetically stable folded structures. Notably, we also observed this stabilizing effect for the folding of the TL-RNA construct. Similar observations supporting the notion that apolar chemical environments stabilize the formation of RNA tertiary structures have also been reported previously (52,53). Based on these findings, we conclude that apolar environments, characterized by dielectric constants less than that of pure water, can promote folding via the following physical mechanism: the low dielectric environment facilitates recruitment of counterions, which leads to more compact unfolded structures that are primed to fold, thereby shifting the equilibrium to favor the folded state.

CONCLUSIONS

In this study, we altered the polarity of the local chemical environment surrounding two model RNA using binary mixtures of water and various organic cosolvents. Our results indicate that apolar environments, characterized by dielectric constants less than that of water, generally compact unfolded RNA molecules. We propose that these apolar environments give rise to enhanced condensation of counterions around the negatively charged phosphate groups of the RNA, which minimizes interphosphate repulsion and allows the unfolded RNA to sample more compact conformations. Indeed, our conclusion is bolstered by a recent study that showed nanoscale hydrophobic environments increase the strength of salt bridges formed in an otherwise aqueous media (54), a phenomenon first discussed with regard to protein folding nearly 70 years ago (55).

Furthermore, we observed that this molecular compaction is also accompanied by an energetic stabilization of folded RNA tertiary structures, presumably reflecting a reduction of the conformation search associated with the folding process. Here, it is of course important to note that environments near the surfaces of proteins are quite apolar, where the value of ε_r is thought to be three to four times lower than that of pure water (16). From this simplified point of view, it is reasonable to expect the local chemical environment around an RNA within a ribonucleoprotein complex may in fact be quite apolar, which, as we have shown, can alter the structural and energetic properties of RNA folding. These dielectric effects associated with apolar environments at the surfaces of proteins may also help explain why the unfolded nucleic acid clients of RNA folding chaperones become significantly more compact after associating with their proteinaceous binding partners (14).

SUPPORTING MATERIAL

Supporting material can be found online at https://doi.org/10.1016/j.bpr.2021.100004.

AUTHOR CONTRIBUTIONS

E.D.H. conceived project, designed experiments, provided samples, analyzed data, and wrote the manuscript. S.M.G. designed experiments, performed experiments, analyzed data, and wrote the manuscript.

DECLARATION OF INTERESTS

The authors declare no competing interests.

ACKNOWLEDGMENTS

The authors acknowledge the following individuals and institutions for their contributions to this research: the other members of the Holmstrom lab, as well as Carey K. Johnson, for their insightful feedback during the preparation of this manuscript, and D. Nettels, B. Schuler, and co-workers for developing Fretica (https://schuler.bioc.uzh.ch/programs/), a user-extendable toolbox for analyzing single-molecule fluorescence data within Mathematica (Wolfram Alpha).

The authors acknowledge the University of Kansas and the National Institutes of Health (P20GM103638 to E.D.H.) for financial support.

REFERENCES

1. Ganser, L. R., M. L. Kelly, ..., H. M. Al-Hashimi. 2019. The roles of structural dynamics in the cellular functions of RNAs. *Nat. Rev. Mol. Cell Biol.* 20:474–489.
2. Leamy, K. A., S. M. Assmann, ..., P. C. Bevilacqua. 2016. Bridging the gap between in vitro and in vivo RNA folding. *Q. Rev. Biophys.* 49:e10.
3. Fischer, N. M., M. D. Polêto, ..., D. van der Spoel. 2018. Influence of Na+ and Mg2+ ions on RNA structures studied with molecular dynamics simulations. *Nucleic Acids Res.* 46:4872–4882.
4. Kolev, S. K., P. S. Petkov, ..., G. N. Vayssilov. 2018. Interaction of Na^+, K^+, Mg^{2+} and Ca^{2+} counter cations with RNA. *Metallomics.* 10:659–678.
5. Fiore, J. L., E. D. Holmstrom, ..., D. J. Nesbitt. 2012. The role of counterion valence and size in GAAA tetraloop-receptor docking/undocking kinetics. *J. Mol. Biol.* 423:198–216.
6. Draper, D. E. 2004. A guide to ions and RNA structure. *RNA.* 10:335–343.
7. Rozov, A., I. Khusainov, ..., G. Yusupova. 2019. Importance of potassium ions for ribosome structure and function revealed by long-wavelength X-ray diffraction. *Nat. Commun.* 10:2519.

8. Holmstrom, E. D., J. L. Fiore, and D. J. Nesbitt. 2012. Thermodynamic origins of monovalent facilitated RNA folding. *Biochemistry.* 51:3732–3743.
9. Bowman, J. C., T. K. Lenz, …, L. D. Williams. 2012. Cations in charge: magnesium ions in RNA folding and catalysis. *Curr. Opin. Struct. Biol.* 22:262–272.
10. Fiore, J. L., E. D. Holmstrom, and D. J. Nesbitt. 2012. Entropic origin of Mg2+-facilitated RNA folding. *Proc. Natl. Acad. Sci. USA.* 109:2902–2907.
11. Cole, P. E., S. K. Yang, and D. M. Crothers. 1972. Conformational changes of transfer ribonucleic acid. Equilibrium phase diagrams. *Biochemistry.* 11:4358–4368.
12. Römer, R., and R. Hach. 1975. tRNA conformation and magnesium binding. A study of a yeast phenylalanine-specific tRNA by a fluorescent indicator and differential melting curves. *Eur. J. Biochem.* 55:271–284.
13. Weeks, K. M. 1997. Protein-facilitated RNA folding. *Curr. Opin. Struct. Biol.* 7:336–342.
14. Holmstrom, E. D., Z. Liu, …, B. Schuler. 2019. Disordered RNA chaperones can enhance nucleic acid folding via local charge screening. *Nat. Commun.* 10:2453.
15. Sharma, R., S. Kk, …, F. Westerlund. 2020. Real-time compaction of nanoconfined DNA by an intrinsically disordered macromolecular counterion. *Biochem. Biophys. Res. Commun.* 533:175–180.
16. Li, L., C. Li, …, E. Alexov. 2013. On the dielectric "constant" of proteins: smooth dielectric function for macromolecular modeling and its implementation in DelPhi. *J. Chem. Theory Comput.* 9:2126–2136.
17. Nakano, S.-i., M. Horita, …, N. Sugimoto. 2017. Catalytic activities of ribozymes and DNAzymes in water and mixed aqueous media. *Catalysts.* 7:355.
18. Nakano, S., Y. Kitagawa, …, N. Sugimoto. 2014. Hammerhead ribozyme activity and oligonucleotide duplex stability in mixed solutions of water and organic compounds. *FEBS Open Bio.* 4:643–650.
19. Nakano, S. I., and N. Sugimoto. 2016. Model studies of the effects of intracellular crowding on nucleic acid interactions. *Mol. Biosyst.* 13:32–41.
20. Nakano, S. I., and N. Sugimoto. 2016. The structural stability and catalytic activity of DNA and RNA oligonucleotides in the presence of organic solvents. *Biophys. Rev.* 8:11–23.
21. Nakano, S., D. Yamaguchi, …, N. Sugimoto. 2012. Hydration changes upon DNA folding studied by osmotic stress experiments. *Biophys. J.* 102:2808–2817.
22. Nakano, S., L. Wu, …, N. Sugimoto. 2008. Conformation and the sodium ion condensation on DNA and RNA structures in the presence of a neutral cosolute as a mimic of the intracellular media. *Mol. Biosyst.* 4:579–588.
23. Nakano, S., H. Karimata, …, N. Sugimoto. 2004. The effect of molecular crowding with nucleotide length and cosolute structure on DNA duplex stability. *J. Am. Chem. Soc.* 126:14330–14331.
24. Nakano, S., H. Hirayama, …, N. Sugimoto. 2012. Dimerization of nucleic acid hairpins in the conditions caused by neutral cosolutes. *J. Phys. Chem. B.* 116:7406–7415.
25. Nakano, S., Y. Kitagawa, …, N. Sugimoto. 2015. Effects of cosolvents on the folding and catalytic activities of the hammerhead ribozyme. *ChemBioChem.* 16:1803–1810.
26. Pomp, D., and J. F. Medrano. 1991. Organic solvents as facilitators of polymerase chain reaction. *Biotechniques.* 10:58–59.
27. Green, M. R., and J. Sambrook. 2020. Precipitation of RNA with ethanol. *Cold Spring Harb. Protoc.* 2020:101717.
28. Chomczynski, P., and N. Sacchi. 2006. The single-step method of RNA isolation by acid guanidinium thiocyanate-phenol-chloroform extraction: twenty-something years on. *Nat. Protoc.* 1:581–585.
29. Azarani, A., and K. H. Hecker. 2001. RNA analysis by ion-pair reversed-phase high performance liquid chromatography. *Nucleic Acids Res.* 29:E7.
30. Akhadov, Y. Y. 1980. Chapter III - Dielectric data for binary systems — aqueous solutions. *In* Dielectric Properties of Binary Solutions. Y. Y. Akhadov, ed. Pergamon, pp. 266–338.
31. Akerlof, G. 1932. Dielectric constants of some organic solvent-water mixtures at various temperatures. *J. Am. Chem. Soc.* 54:4125–4139.
32. Gagliardi, L. G., C. B. Castells, …, E. Bosch. 2007. Static dielectric constants of acetonitrile/water mixtures at different temperatures and Debye–Hückel A and a0B parameters for activity coefficients. *J. Chem. Eng. Data.* 52:1103–1107.
33. Fiore, J. L., and D. J. Nesbitt. 2013. An RNA folding motif: GNRA tetraloop-receptor interactions. *Q. Rev. Biophys.* 46:223–264.
34. Katritzky, A. R., D. C. Fara, …, M. Karelson. 2004. Quantitative measures of solvent polarity. *Chem. Rev.* 104:175–198.
35. Sen, A., and P. E. Nielsen. 2007. On the stability of peptide nucleic acid duplexes in the presence of organic solvents. *Nucleic Acids Res.* 35:3367–3374.
36. Roy, R., S. Hohng, and T. Ha. 2008. A practical guide to single-molecule FRET. *Nat. Methods.* 5:507–516.
37. Lerner, E., T. Cordes, …, S. Weiss. 2018. Toward dynamic structural biology: two decades of single-molecule Förster resonance energy transfer. *Science.* 359:eaan1133.
38. Hodak, J. H., C. D. Downey, …, D. J. Nesbitt. 2005. Docking kinetics and equilibrium of a GAAA tetraloop-receptor motif probed by single-molecule FRET. *Proc. Natl. Acad. Sci. USA.* 102:10505–10510.
39. Kapanidis, A., D. Majumdar, …, S. Weiss. 2015. Alternating laser excitation for solution-based single-molecule FRET. *Cold Spring Harb. Protoc.* 2015:979–987.
40. Schuler, B., and D. Nettels. 2020. Fretica https://schuler.bioc.uzh.ch/programs/.
41. Holmstrom, E. D., A. Holla, …, B. Schuler. 2018. Accurate transfer efficiencies, distance distributions, and ensembles of unfolded and intrinsically disordered proteins from single-molecule FRET. *Methods Enzymol.* 611:287–325.
42. Laurence, C., S. Mansour, …, J. Legros. 2021. Hydrogen-bond acceptance of solvents: a ^{19}F solvatomagnetic β_1 database to replace solvatochromic and solvatovibrational scales. *J. Org. Chem.* 86:4143–4158.
43. Hefter, G., Y. Marcus, and W. E. Waghorne. 2002. Enthalpies and entropies of transfer of electrolytes and ions from water to mixed aqueous organic solvents. *Chem. Rev.* 102:2773–2836.
44. Fiore, J. L., J. H. Hodak, …, D. J. Nesbitt. 2008. Monovalent and divalent promoted GAAA tetraloop-receptor tertiary interactions from freely diffusing single-molecule studies. *Biophys. J.* 95:3892–3905.
45. Batey, R. T., R. P. Rambo, and J. A. Doudna. 1999. Tertiary motifs in RNA structure and folding. *Angew. Chem. Int. Ed. Engl.* 38:2326–2343.
46. Brion, P., and E. Westhof. 1997. Hierarchy and dynamics of RNA folding. *Annu. Rev. Biophys. Biomol. Struct.* 26:113–137.
47. Feng, B., R. P. Sosa, …, B. Nordén. 2019. Hydrophobic catalysis and a potential biological role of DNA unstacking induced by environment effects. *Proc. Natl. Acad. Sci. USA.* 116:17169–17174.
48. Draper, D. E. 2008. RNA folding: thermodynamic and molecular descriptions of the roles of ions. *Biophys. J.* 95:5489–5495.
49. Woodson, S. A. 2010. Compact intermediates in RNA folding. *Annu. Rev. Biophys.* 39:61–77.
50. Bonilla, S., C. Limouse, …, D. Herschlag. 2017. Single-molecule fluorescence reveals commonalities and distinctions among natural and in vitro-selected RNA tertiary motifs in a multistep folding pathway. *J. Am. Chem. Soc.* 139:18576–18589.
51. Russell, R., I. S. Millett, …, L. Pollack. 2002. Rapid compaction during RNA folding. *Proc. Natl. Acad. Sci. USA.* 99:4266–4271.

52. Shiman, R., and D. E. Draper. 2000. Stabilization of RNA tertiary structure by monovalent cations. *J. Mol. Biol.* 302:79–91.

53. Strulson, C. A., J. A. Boyer, …, P. C. Bevilacqua. 2014. Molecular crowders and cosolutes promote folding cooperativity of RNA under physiological ionic conditions. *RNA.* 20:331–347.

54. Chen, S., Y. Itoh, …, T. Aida. 2015. Ionic interactions. Subnanoscale hydrophobic modulation of salt bridges in aqueous media. *Science.* 348:555–559.

55. Schellman, J. A. 1953. The application of the Bjerrum ion association theory to the binding of anions by proteins. *J. Phys. Chem.* 57:472–475.

Biophysical Reports, Volume *1*

Supplemental information

Apolar chemical environments compact unfolded RNAs and can promote folding

Shamal M. Gunawardhana and Erik D. Holmstrom

Apolar chemical environments compact unfolded RNAs and can promote folding

Compaction of RNA in apolar environments (running title)

Shamal M. Gunawardhana[1] and Erik D. Holmstrom[1,2]*

[1]Department of Molecular Biosciences, University of Kansas, Lawrence, Kansas, USA
[2]Department of Chemistry, University of Kansas, Lawrence, Kansas, USA

* To whom correspondence should be addressed. Tel: +1 (785) 864 1712; Email: erik.d.holmstrom@ku.edu

SUPPLEMENTAL INFORMATION

Fundamentally, the efficiency (E) of Förster resonance energy transfer in a FRET experiment depends on two critical parameters (Eq. S1), both of which have units of distance. The first parameter is the physical nanoscopic distance, r, separating the dipoles of the two interacting fluorophores. The second parameter, called the Förster distance (R_0), is a numerical quantity (Eq. S2) that specifically characterizes the two interacting fluorophores. The exact value depends on several factors, including: (*i*) the relative spatial orientation of the two interacting dipoles, which is symbolized via κ^2; (*ii*) the fluorescence quantum yield of the donor fluorophore in the absences of the acceptor fluorophore, which is symbolized via Q_D; (*iii*) the overlap integral between the donor emission spectrum and the acceptor absorption spectrum, which is symbolized via J; and (*iv*) the refractive index of the medium surrounding the fluorophores, symbolized via n_D (1).

$$E = R_0^6 / (R_0^6 + r^6) \qquad \text{Eq. S1}$$

$$R_0^6 = \frac{9000 \ln(10)\, \kappa^2\, Q_D\, J}{128\, \pi^5 n_D^4\, N_A} \qquad \text{Eq. S2}$$

The numerical value of R_0 is on the order of 5 nm, depending on the identity of donor and acceptor fluorophores, and is often assumed to be constant throughout an experiment. In this way, any experimentally observed changes in E can be attributed to changes in the inter-fluorophore distance, r. However, given that the value of R_0 itself depends on several factors, it is possible that experimentally observed changes in E may result from changes in R_0 as well as r.

For example, the refractive index of our binary solvent mixtures is not identical to pure water. However, the values of n_D for these cosolvents (Table S1) do not substantially deviate from that of a purely aqueous solution. From Eq. S2, one can see that the R_0 is proportional to $n_D^{-2/3}$. Therefore, small changes to the value of n_D will barely alter the value of R_0, which, alone, would not be enough to account for the experimentally observed changes in the value of $\langle E \rangle$.

Solvent	n_D	R_0 (solvent) / R_0(water)
H_2O	1.3330	1.0000
MeOH *	1.3287	1.0022
EtOH *	1.3615	0.9860
IPA *	1.3776	0.9783
ACN *	1.3427	0.9952
DMK ‡	1.3598	0.9868

Table S1: Index of refraction (n_D) values at a wavelength that corresponds to the emission of the donor fluorophore (i.e., 570 nm) for the solvents used in this report. The relationship between R_0 and n_D (Eq, S2) is used to calculate the expected effect on the Förster distance. The * denotes values taken from (2) and the ‡ denotes values taken from (3).

Additionally, it is well known that organic solvents can also influence the photophysical properties of fluorophores (4), including: (*i*) the fluorescence lifetime, τ_{fl}, and (*ii*) the rotational correlation time, τ_{rot}. Importantly, solvent-dependent changes in τ_{fl} and τ_{rot} can alter the value of R_0 via Q_D and κ^2, respectively. To determine how our organic cosolvents influence the values of τ_{fl} and τ_{rot} for the donor and acceptor fluorophores additional measurements were performed using interleaved pulsed excitation (5). These experiments were performed on the same microscope system as the alternating continuous-wave excitation experiments described in the main text, however the two dual-mode lasers were set to output ~ 100 µW of short (< 150 ps) pulses at a repetition rate of 20 MHz, which allowed us to lower the temporal resolution of the TCSPC module by more than three orders of magnitude to a value of 16 ps (i.e., 2^4 ps). Pulsed excitation experiments were conducted on samples containing either water as the only solvent or binary aqueous-organic solvent mixtures at 45% (v/v). In either case, all samples contained 250 mM of KCl, 25 mM HEPES, 12.5 mM NaOH and 0.01% tween 20 (v/v). Fluorescence intensity decay (FID) histograms were constructed from the TCSPC data for the donor-only ($S > 0.9$) and acceptor-only ($S < 0.1$) sub-populations of fluorescently labelled molecules. The FID histograms were then tail-fit using a single decaying exponential function (6) to estimate the fluorescence lifetime of the donor (${}^{Don}\tau_{fl}$) and acceptor (${}^{Acc}\tau_{fl}$) fluorophores (Table S2).

HP-RNA				
**Solvent (v/v)**	$^{Don}\tau_{fl}$ (ns)	$^{Acc}\tau_{fl}$ (ns)	$^{Don}\tau_{rot}$ (ns)	$^{Acc}\tau_{rot}$ (ns)
100% H_2O	2.72 ± 0.02	2.97 ± 0.01	0.84 ± 0.22	0.62 ± 0.12
45% MeOH	2.72	3.15	0.85	0.99
45% EtOH	2.68	3.21	0.92	1.18
45% IPA	2.67	3.23	1.04	1.35
45% ACN	2.62	3.41	0.70	0.64
45% DMK	2.66	3.26	0.86	0.84
**TL-RNA**				
**Solvent (v/v)**	$^{Don}\tau_{fl}$ (ns)	$^{Acc}\tau_{fl}$ (ns)	$^{Don}\tau_{rot}$ (ns)	$^{Acc}\tau_{rot}$ (ns)
100% H_2O	2.68	2.93	0.90	0.49
45% MeOH	2.66	3.14	1.14	0.53
45% EtOH	2.65	3.19	1.15	0.60
45% IPA	2.64	3.21	1.49	0.70
45% ACN	2.66	3.40	0.66	0.40
45% DMK	2.64	3.25	0.96	0.46

Table S2: Fluorescence lifetime and rotational correlation time of the donor and acceptor fluorophores under various experimental conditions (100 pM RNA, 25 mM HEPES, 12.5 mM NaOH, 250 mM KCl, 0.01% v/v Tween 20, and the specified amount of organic solvent).

In general, the organic cosolvents slightly decreased (< 4%) the fluorescence lifetime of the donor fluorophore, ${}^{Don}\tau_{fl}$. If this slight decrease in lifetime was entirely due to non-radiative processes, then the donor quantum yield (Q_D) would also decrease by < 4%. From Eq. S2, one can see that R_0 is proportional to the sixth-root of Q_D. As such, a 4% decrease in Q_D would have little effect (< 1%) on the R_0 and therefore it would not be expected to substantially change the resulting FRET efficiency. For these reasons, any experimentally observed changes in the value of E in apolar conditions are likely due to changes in the inter-fluorophore distance, r, rather than R_0.

The fluorescence lifetime of the acceptor fluorophore, ${}^{Acc}\tau_{fl}$, is slightly more influenced by the presence of organic cosolvents and increases by as much as 16%. Fortunately, ${}^{Acc}\tau_{fl}$ does not influence the value of R_0 and can be accounted for when accurately determining transfer efficiencies via the correction factor associated with the non-identical detection efficiencies of the donor and acceptor fluorophores.

Different solvents can also influence the rotational motion of the fluorophores, leading to changes in the rotational correlation time, τ_{rot}, which can affect the value of κ^2 associated with R_0. Unfortunately, direct measurements of κ^2 are challenging and therefore a value of κ^2 = 2/3 is often used, which corresponds to the dynamical average of two fluorophores, each sampling an isotropic distribution of orientations on a time scale that is much faster than τ_{fl}. However, the assumed value of 2/3 may not be appropriate in every situation and the true value of κ^2 may change with changing experimental conditions.

Fortunately, the rotational correlation time is much easier to experimentally measure and can be used to determine upper and lower limits on the value of κ^2. To accomplish this, the parallel and perpendicular components of the FID histograms were used to construct fluorescence anisotropy decay (FAD) histograms, which were tail-fit using a single decaying exponential function (7) to estimate the rotational correlation time of the donor (${}^{Don}\tau_{rot}$) and acceptor (${}^{Acc}\tau_{rot}$) fluorophores coupled to the RNA of interest (Table S2). In all cases, the rotational correlation times were always lower in magnitude than the fluorescence lifetimes, suggesting that the two fluorophores were rapidly tumbling in solution. From here, τ_{rot} is used in conjunction with the Perrin equation (8), $(r_{ss}/r_0) = (1+(\tau_{fl} / \tau_{rot}))^{-1}$, to estimate the depolarization factor for the donor (${}^{Don}d_x$) and acceptor (${}^{Acc}d_x$) fluorophores (9), where $d_x = (r_{ss} / r_0)^{1/2}$, with r_{ss} and r_0 representing the steady-state and fundamental fluorescence anisotropies of the fluorophore. Next, the depolarization factors are used to calculate the limiting values of κ^2 (9). With these numbers in hand, we can then determine minimum and maximum values of R_0 relative to a value of R_0 assuming κ^2 = 2/3 (Table S3).

Such an analysis indicates that these solvent-dependent changes in rotational motion of the fluorophore may slightly alter the range of possible values associated with the Förster distances. However, even in the extreme limiting cases the magnitude of this effect is only 10-15%, which would suggest that the observed changes in the experimentally measured transfer efficiency likely arise from changes in inter-fluorophore distance (e.g., biomolecular conformational changes) rather than photophysical changes that would alter the value of R_0.

HP-RNA				
**Solvent (v/v)**	$^{Don}d_x$	$^{Acc}d_x$	$^{min}R_0/R_0$	$^{max}R_0/R_0$
100% H_2O	0.486	0.416	0.905	1.166
45% MeOH	0.488	0.489	0.894	1.180
45% EtOH	0.506	0.518	0.887	1.188
45% IPA	0.529	0.543	0.880	1.197
45% ACN	0.459	0.398	0.911	1.157
45% DMK	0.494	0.453	0.899	1.174
**TL-RNA**				
**Solvent (v/v)**	$^{Don}d_x$	$^{Acc}d_x$	$^{min}R_0/R_0$	$^{max}R_0/R_0$
100% H_2O	0.501	0.379	0.908	1.161
45% MeOH	0.548	0.380	0.901	1.169
45% EtOH	0.550	0.398	0.898	1.173
45% IPA	0.601	0.423	0.887	1.186
45% ACN	0.446	0.324	0.922	1.141
45% DMK	0.516	0.352	0.909	1.158

Table S3: Depolarization factors for the donor and acceptor fluorophores determined using the Perrin Equation and the fluorescence lifetime and rotational correlation time of the respective fluorophores attached to the RNA of interest. Depolarization factors are used to estimate minimum and maximum values for the orientation factor (κ^2), which are then used to calculate upper and lower bounds for the characteristic Förster distance (R_0) in the binary aqueous-organic solvent mixtures (100 pM RNA, 25 mM HEPES, 12.5 mM NaOH, 250 mM KCl, 0.01% v/v Tween 20, and the specified amount of organic solvent).

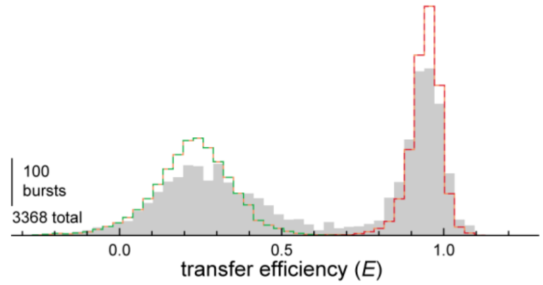

Figure S1: Near shot noise-limited width of single-molecule transfer efficiency histograms. Photons from bursts with transfer efficiency values greater than or less than 0.7 were used to generate shot noise-limited histograms for the folded (red) and unfolded sub-populations (green). Such an analysis assumes that true distribution of transfer efficiencies associated with the two sub-populations is infinitely narrow, with values for the mean transfer efficiency, $\langle E \rangle$, and fractional abundance, Θ, of the folded and unfolded sub-populations fixed at those determined via the gaussian fitting procedure described in the main text. The sum of these shot noise-limited histograms (orange) is only slightly narrower than the experimentally observed histograms (gray) recorded under typical solution conditions (100 pM RNA, 25 mM HEPES, 12.5 mM NaOH, 250 mM KCl, 0.01% v/v Tween 20). The excess width associated with the experimental histograms could indicate a small amount of conformational heterogeneity within the folded and unfolded sub-populations.

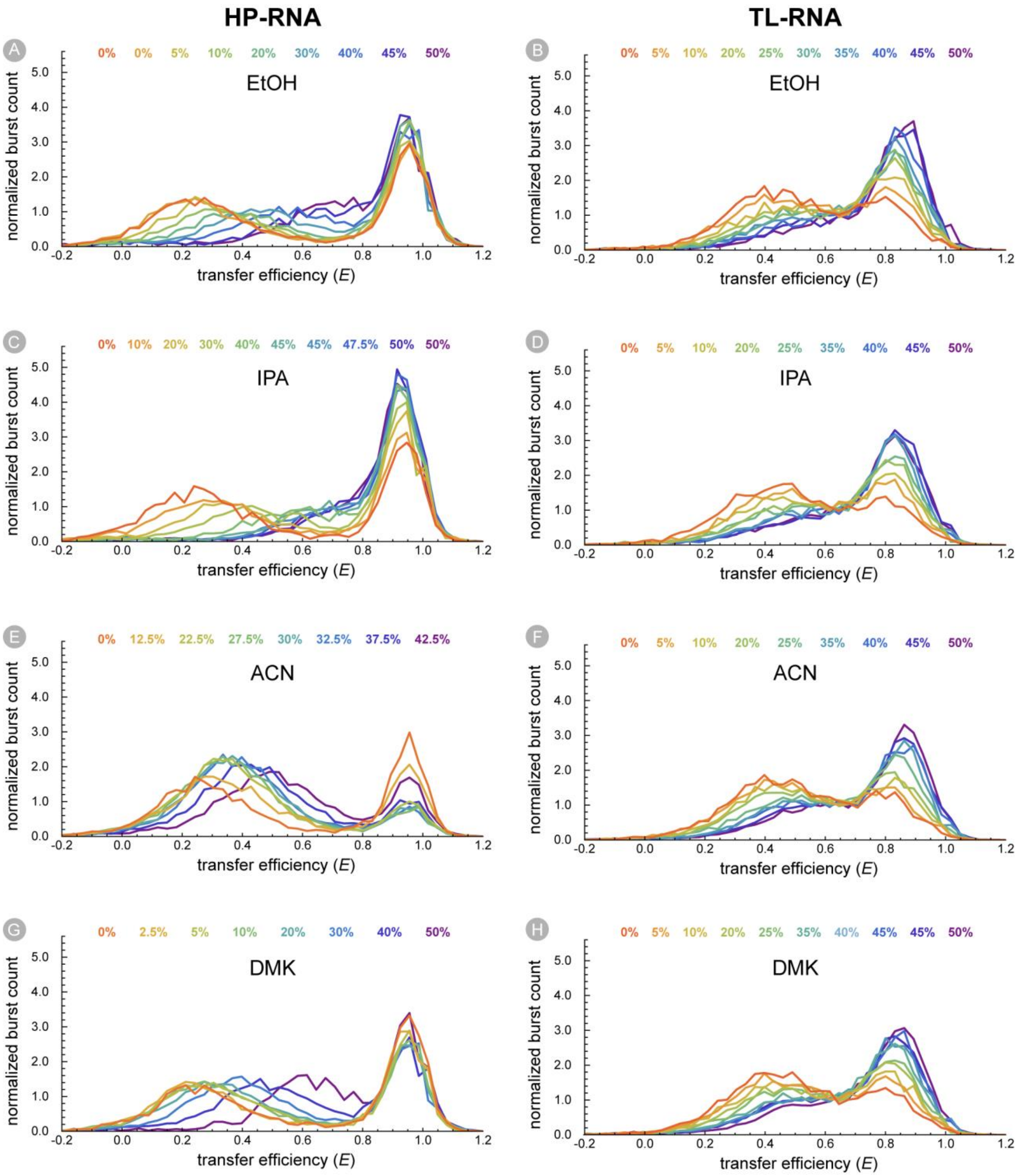

Figure S2: Transfer efficiency histograms of RNAs in apolar chemical environments. Transfer efficiency histograms are shown for the HP-RNA (left) and TL-RNA (right) in binary aqueous organic mixtures consisting of water and various amounts of EtOH **(A,B)**, IPA **(C,D)**, ACN **(E,F)**, or DMK **(G,H)**. Solution conditions were as follows: (100 pM RNA, 25 mM HEPES, 12.5 mM NaOH, 250 mM KCl, 0.01% v/v Tween 20, and the specified amount organic solvent) Note: some histograms reflect measurements that were performed under identical experimental conditions.

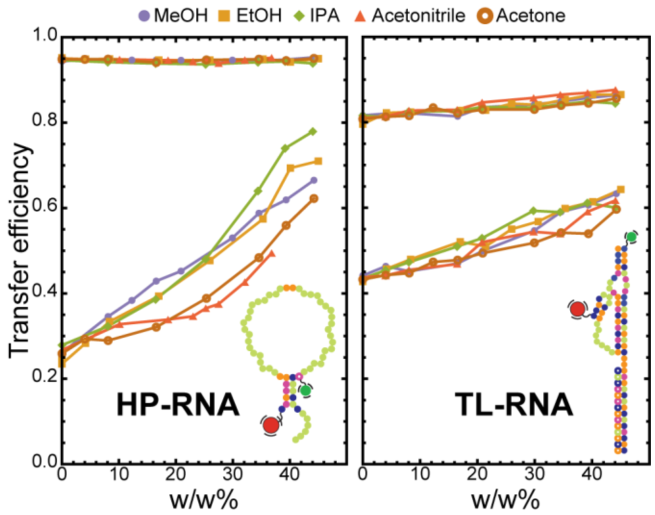

Figure S3: Polarity of local chemical environment modulates the dimensions of structured RNAs. This figure displays the information depicted in Figure 4A and 4B of main text. However, the x-axis represents the amount (in weight percent) of organic cosolute in the sample solutions instead of the relative dielectric constant, ε_r, of that cosolvent system (100 pM RNA, 25 mM HEPES, 12.5 mM NaOH, 250 mM KCl, 0.01% v/v Tween 20, and the specified amount of organic solvent).

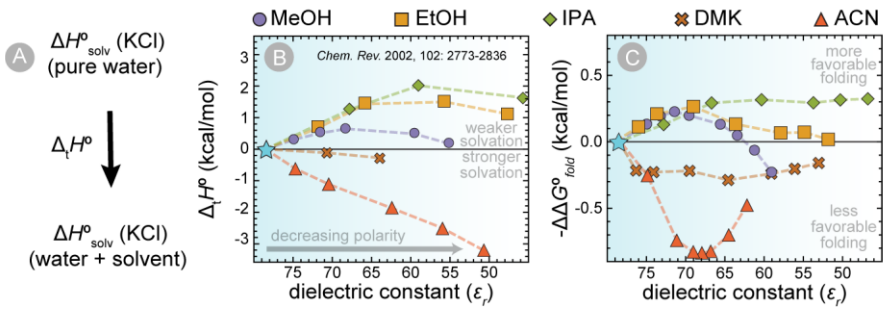

Figure S4: Solvent Transfer Enthalpies for KCl. (A) Solvent transfer enthalpy ($\Delta_t H°$) measures the change in the solvation enthalpy ($\Delta H°_{solv}$) that occurs when an electrolyte (e.g., KCl) is transferred from pure water another solvent system (e.g., a binary aqueous organic mixture). It provides an energetic measure of how the solvation of the electrolyte changes, with larger positive values indicating weaker solvation. **(B)** A plot of the reported values (10) of $\Delta_t H°$ associated with transferring KCl from water to the aqueous organic mixtures used in this study. The solvents ACN and DMK strengthen the solvation of KCl, whereas the protic alcohols, IPA EtOH and MeOH, non-monotonically weaken the solvation of KCl. **(C)** Negative values of $\Delta\Delta G°_{fold}$ qualitatively mirror the values of $\Delta_t H°$, suggesting that folding of the HP-RNA becomes more favorable when the solvation of the dominant electrolyte (e.g., KCl) is less enthalpically favorable.

1. Schuler, B. 2013. Single-molecule FRET of protein structure and dynamics - a primer. *J Nanobiotechnology.* 11 Suppl 1:S2, doi: 10.1186/1477-3155-11-S1-S2.

2. Kozma, I. Z., P. Krok, and E. Riedle. 2005. Direct measurement of the group-velocity mismatch and derivation of the refractive-index dispersion for a variety of solvents in the ultraviolet. *J Opt Soc Am B.* 22:1479-1485, doi: Doi 10.1364/Josab.22.001479.

3. Rheims, J., J. Koser, and T. Wriedt. 1997. Refractive-index measurements in the near-IR using an Abbe refractometer. *Meas Sci Technol.* 8:601-605, doi: Doi 10.1088/0957-0233/8/6/003.

4. Anonymous, "Solvent and Environmental Effects" in Principles of Fluorescence Spectroscopy, Lakowicz, J. R., Ed. (Springer US, Boston, MA, 2006), 10.1007/978-0-387-46312-4_6, pp. 205-235.

5. Hendrix, J., and D. C. Lamb. 2013. Pulsed interleaved excitation: principles and applications. *Methods Enzymol.* 518:205-243, doi: 10.1016/B978-0-12-388422-0.00009-1.

6. Anonymous, "Time-Domain Lifetime Measurements" in Principles of Fluorescence Spectroscopy, Lakowicz, J. R., Ed. (Springer US, Boston, MA, 2006), 10.1007/978-0-387-46312-4_4, pp. 97-155.

7. Anonymous, "Time-Dependent Anisotropy Decays" in Principles of Fluorescence Spectroscopy, Lakowicz, J. R., Ed. (Springer US, Boston, MA, 2006), 10.1007/978-0-387-46312-4_11, pp. 383-412.

8. Anonymous, "Fluorescence Anisotropy" in Principles of Fluorescence Spectroscopy, Lakowicz, J. R., Ed. (Springer US, Boston, MA, 2006), 10.1007/978-0-387-46312-4_10, pp. 353-382.

9. Anonymous, "Energy Transfer" in Principles of Fluorescence Spectroscopy, Lakowicz, J. R., Ed. (Springer US, Boston, MA, 2006), 10.1007/978-0-387-46312-4_13, pp. 443-475.

10. Hefter, G., Y. Marcus, and W. E. Waghorne. 2002. Enthalpies and entropies of transfer of electrolytes and ions from water to mixed aqueous organic solvents. *Chem Rev.* 102:2773-2836, doi: 10.1021/cr010031s.
